# Deciphering the functional roles of PE18 and PPE26 proteins in modulating *Mycobacterium tuberculosis* pathogenesis and immune response

**DOI:** 10.3389/fimmu.2025.1517822

**Published:** 2025-01-30

**Authors:** Aquib Ehtram, Mohd Shariq, Neha Quadir, Salma Jamal, Manjunath Pichipalli, Sheeba Zarin, Javaid Ahmad Sheikh, Nasreen Z. Ehtesham, Seyed E. Hasnain

**Affiliations:** ^1^ Kusuma School of Biological Sciences, Indian Institute of Technology Delhi, New Delhi, India; ^2^ GITAM School of Science, Gandhi Institute of Technology and Management (GITAM) University, Hyderabad, Telangana, India; ^3^ Inflammation Biology and Cell Signaling Laboratory, ICMR-National Institute of Pathology, New Delhi, India; ^4^ Jamia Hamdard Institute of Molecular Medicine, Jamia Hamdard, New Delhi, India; ^5^ Department of Life Science, School of Basic Sciences and Research, Sharda University, Greater Noida, Uttar Pradesh, India; ^6^ Department of Biotechnology, Jamia Hamdard, New Delhi, India; ^7^ Department of Biochemical Engineering and Biotechnology, Indian Institute of Technology Delhi, New Delhi, India

**Keywords:** macrophage activation marker, phagosome, host-pathogen interaction, Th1 immune response, immune modulation, proinflammatory cytokine, IL-1 beta, IL-6

## Abstract

**Introduction:**

Tuberculosis (TB), caused by *Mycobacterium tuberculosis* (Mtb), remains a leading cause of mortality worldwide. A crucial factor in *Mtb's* virulence is the ESX-5 secretion system, which transports PE/PPE proteins such as PE18 and PPE26. These proteins modulate host-pathogen interactions, immune responses, and intracellular survival mechanisms. Despite their importance, the roles and molecular interactions of PE18 and PPE26 in *Mtb* pathogenesis require further investigation.

**Methods:**

We explored the roles of PE18 and PPE26 using recombinant *Mycobacterium smegmatis* (*Msmeg*) as a model organism. Protein-protein interactions were analyzed biochemically to identify partners within the ESX-5 secretion system, including EspG5 and other PE/PPE proteins. Subcellular localization of these proteins was assessed via cell fractionation studies. Functional assays, including *in vitro* cytokine production and antigen presentation studies, were performed using TLR2/Myd88 knockout and wild-type macrophages. *In vivo* experiments were conducted to assess effector T-cell activation and intracellular survival. Mechanistic insights into endosome-phagosome maturation and actin cytoskeleton dynamics were obtained through fluorescence microscopy.

**Results:**

Our biochemical analyses confirmed interactions between PE18/PPE26, PE18/PPE27, PE19/PPE25, and EspG5/PPE, highlighting their involvement in ESX-5-mediated secretion. Cell fractionation studies revealed that PE/PPE proteins predominantly localize to the cell wall, with PE18 also secreted extracellularly. In vitro and *in vivo* experiments demonstrated that PE18 and PPE26 activate cytokine production and antigen presentation via TLR2/Myd88-dependent signaling pathways, inducing robust effector memory T-cell responses. Recombinant *Msmeg* expressing PE18, PPE26, or their combination exhibited enhanced intracellular survival by disrupting endosome-phagosome maturation, likely through interference with actin cytoskeletal organization.

**Discussion:**

Our findings elucidate the pivotal roles of PE18 and PPE26 in *Mtb* pathogenesis, emphasizing their contributions to immune modulation and intracellular persistence. The observed disruption of actin dynamics and endosome-phagosome maturation underscores a novel mechanism by which *Mtb* evades host defenses. The ability of PE18 and PPE26 to induce effector T-cell responses highlights their potential as targets for host-directed therapies or vaccine development against TB. Further studies focusing on their structure-function relationships and interactions with host proteins could accelerate the development of innovative therapeutic strategies.

## Highlights

- PE18 and PPE26 interact physically, indicating synergy in immune modulation.
- PE18 is a cell wall-associated secretory protein in *Mycobacterium tuberculosis*.
- PE18 and PPE26 trigger proinflammatory cytokines, promoting T-cell activation.
- Recombinant *Mycobacterium smegmatis* expressing PE18/PPE26 shows enhanced survival in macrophage cells.
- PE18 and PPE26 inhibit actin-mediated cytoskeletal changes, impeding phagosome maturation.

## 1 Introduction

*Mtb* causes TB, one of the deadliest infectious diseases (1, 2). Although the etiological agent was identified more than a century ago, the disease-causing mechanisms remain yet elusive (3). *Mtb* employs an array of effector proteins for successful virulence and pathogenesis, culminating in colonizing the host (4–9). Intriguingly, *Mtb* harbors a unique family of proteins known as the PE/PPE family, which is exclusively found within the genus *Mycobacterium* and absent in all other organisms across the living kingdom. This protein family is named for its characteristic Proline-Glutamic acid (PE) and Proline-Proline-Glutamic acid (PPE) conserved motifs in N terminal domain. Almost 7-10% of the genome is devoted to express ∼99 PE and ∼60 PPE genes. Based on the highly polymorphic C terminal domain, these families are further subdivided into various subfamilies like, PE-PGRS (polymorphic GC-rich-sequence) and PPE-MPTR (major polymorphic tandem repeat) etc. The observation that PE and PPE family of proteins are selectively present in pathogenic mycobacterial species, suggests their critical role in pathogenesis and virulence (5, 10–13). PE/PPE proteins are crucial protein factors exploited by *Mtb* against the host and are responsible for various pro-pathogen functions (5, 12, 14–16). Being primarily localized on cell wall or secreted out, thus directly placed at host pathogen interface, these proteins are mainly involved in antigenic diversity, immune evasion and pathogenicity (17). Apart from evoking cellular and humoral immunity, these proteins have been implicated in regulating virulence by altering the transport of major virulence factors (18). These proteins also regulate cell death pathways along with key homeostatic pathways, such as autophagy that are vital for clearance of intracellular pathogens (11, 19). Emerging evidences suggest that these proteins form solute specific channels in the otherwise impermeable mycomembrane akin to bacterial porins involved in nutrient uptake implying them as potent drug targets (12, 20, 21).

*Mtb* uses type VII secretion systems (T7SSs) to transport virulence-causing proteins, including most PE and PPE proteins, to the bacterial surface and into the host cells to manipulate host-pathogen interactions (22–24). Among the T7SSs of *Mtb*, ESX-5 is a recently evolved secretory apparatus specific to pathogenic mycobacteria (6, 25). The *pe/ppe* genes*, pe18, pe19, ppe25, ppe26, and ppe27*, exist as a single cluster within the ESX-5 genetic locus. Previous studies have demonstrated that PE/PPE protein heterodimers, homodimers, and monomers are secreted into the outer cell envelope of *Mtb*, which interacts with toll-like receptors (TLRs) such as TLR2 or TLR4, which ultimately govern the function of the host immune system (26–29). PE/PPE proteins mainly exist as heterodimers and may exhibit specialized functions different from those of individual proteins *per se* (30). These heterodimers likely interact with ESX-5–encoded chaperone EspG5 for secretion through ESX-5 secretion system as reported for PE25–PPE41 (13). EspG5 usually binds PPE partner of the heterodimer without affecting the structure of PE/PPE pair. Crystal structure studies suggest that EspG5 acts as a signal recognition particle that binds to newly synthesized PE/PPE protein for their transport through ESX-5 (31). This chaperone itself is not secreted but gets dissociated from the PPE/PE-heterodimer at the membrane (32). It has been shown that genetic disruption of *pe18, pe19, ppe26, ppe27*, and Δ*ppe27-pe19* diminished the virulence capability of *Mtb* in the mouse model, suggesting a crucial role played by these proteins in pathogenesis (33, 34). Disruption of the ESX-5 locus severely hampers the secretion of PE and PPE family proteins in *Mtb* (33). Furthermore, the PE/PPE proteins of *Mtb* are integral to its immunomodulatory strategies, enabling evasion of host defenses and promoting chronic infection. These proteins influence immune responses through diverse mechanisms. For instance, PPE18 has been shown to inhibit macrophage activation by reducing pro-inflammatory cytokines like TNFα and IL12, thereby impairing effective immune signaling. Similarly, PE_PGRS47 prevents phagosome-lysosome fusion, a critical process for bacterial clearance, enhancing intracellular survival (18, 26). PE_PGRS47 also inhibits autophagy initiation and MHCII antigen presentation, thereby, dampening protective host immune responses (35, 36). Furthermore, PE_PGRS62 has been implicated in downregulating apoptosis pathways, promoting a permissive intracellular environment for bacterial persistence (37). On the other hand, PE13 activates the p38-MAPK pathway, driving macrophage apoptosis and facilitating bacterial dissemination (38). These examples highlight the sophisticated role of PE/PPE proteins in manipulating host immunity, highlighting their importance as therapeutic and diagnostic targets.

Therefore, it is worth exploring the critical roles of PE18 and PPE26 proteins of *Mtb* in immune function regulation and virulence. Although, the ESX-5 locus encodes multiple PE/PPE proteins such as PE18, PE19, PPE25, PPE26, and PPE27 however, unlike PE18 and PPE26, other PE/PPE proteins within this locus are not encoded in a continuous stretch of DNA. Our decision to focus on PE18 and PPE26 is based on their organization as part of a single operon, suggesting a functional linkage. This operonic arrangement implies that PE18 and PPE26 are co-regulated and likely function together in immune modulation and host-pathogen interactions.

Using *in vitro* and *in vivo* models, we demonstrated that PE18 and PPE26 are co-transcribed in *Mtb* and exhibit cooperative functions in modulating immune responses and host-pathogen interactions. Specifically, we characterized their immunomodulatory effects on macrophages and T-cells, revealing their critical roles in regulating immune cell functions. Our findings highlight that PE18 and PPE26 influence cytoskeletal dynamics, impairing phagolysosomal fusion and thereby enhancing bacterial persistence. Moreover, we explored their contributions to virulence, further elucidating their significance in the pathogenesis of *Mtb*. Notably, the suppression of actin-mediated cytoskeletal rearrangements and inhibition of endosome-phagosome maturation were identified as key mechanisms by which PE18 and PPE26 facilitate bacterial survival and immune evasion. In summary, this research highlights the crucial functions of PE18 and PPE26 in modulating immune responses and influencing cytoskeletal dynamics. These findings provide significant insights into the disease mechanisms of TB, particularly in relation to the *pe/ppe* gene family.

## 2 Materials and methods

### 2.1 *In-silico* interactome prediction of ESX-5 associated PE/PPE proteins

The ClusPro server (https://cluspro.org) was used for docking the modeled PE18 (Rv1788), PPE25 (Rv1787), PPE26 (Rv1789), PE19 (Rv1791), PPE27 (Rv1790), and EspG5 (Rv1794) to determine protein-protein interaction (39–41). The STRING protein-protein interaction database (http://string-db.org) was used to predict the functional association of the protein networks.

### 2.2 Generation of recombinant DNA constructs, protein purification, and recombinant *Msmeg* strains

The *pe18*, *pe19*, *ppe25*, *ppe26*, *ppe27*, and *espG5* genes of *Mtb* were cloned into the pET-28a and pGEX-6P2 expression vectors. Additionally, *pe18* and *ppe26* were cloned into the *E. coli*-*Mtb* shuttle vector pST-2K as both single and double inserts (*pe18+ppe26*). Genomic DNA extracted from *Mtb* was used as the template for gene amplification, with forward and reverse primers containing *EcoRI* and *HindIII* restriction enzyme sites, respectively. The primers used for amplification are listed in **Supplementary Table S1**. Recombinant proteins were expressed in ClearColi^®^ BL21 (DE3) cells (Lucigen, USA) using constructs in the pET-28a vector, resulting in His-tagged proteins. Protein purification was performed using Ni-NTA affinity chromatography in Tris-HCl buffer, followed by overnight dialysis at 4°C using dialysis buffer, as described previously (11, 42). The purity of the recombinant proteins was assessed through SDS-PAGE and western blot analyses (**Supplementary Figures S1A–D**). Residual endotoxin contamination in the purified proteins was removed using polymyxin B-agarose beads, following established protocols (28). The absence of endotoxin was confirmed using the Limulus amoebocyte lysate assay (Thermo Fisher Scientific, USA), performed as per the manufacturer’s instructions (34). Notably, ClearColi^®^ BL21 (DE3) cells are engineered to produce lipopolysaccharide (LPS) with minimal endotoxin activity.

The wild-type *Mycobacterium smegmatis* (*Msmeg_*WT) strain was cultured in 7H9 medium until it reached an optical density (OD600_nm_) of 0.8. Competent cells were prepared using 10% glycerol, following established protocols (43). The *Msmeg_WT* culture was transformed with the pST-2K vector containing *pe18*, *ppe26*, or *pe18+ppe26* genes (1 µg/µl DNA) and incubated at room temperature for 20 min. Electroporation was performed at 2400 V and 30 µF, yielding a pulse duration of 11.2 milliseconds, as described previously (44). Following electroporation, the cells were incubated at 37°C in a shaking incubator for 48 h before being plated onto Middlebrook 7H11 agar supplemented with OADC and 20 µg/ml kanamycin. After 4 days of incubation, colonies of recombinant *Msmeg* expressing *pe18*, *ppe26*, or *pe18+ppe26* were obtained. Recombinant *Msmeg* strains were subsequently cultured in 7H9 medium containing kanamycin. Protein samples were prepared from the recombinant cells, and the expression of target proteins was confirmed via western blot analysis using polyclonal antibodies raised against the proteins (**Supplementary Figure S2A**). Wild-type and recombinant *Msmeg* strains were used to infect macrophages at a multiplicity of infection (MOI) of 1:10 to assess functional outcomes.

### 2.3 Wild-type (RAW264.7) and various TLRs mutant macrophage culture condition

The Biodefense and Emerging Infections Research (BEI) resources (NIAID, NIH) provided the murine macrophage TLR knockout cell lines ΔTLR2, ΔTLR4, ΔTRIF, and ΔMyd88. These cell lines have been derived using primary bone marrow cells from TLR/TRIF/Myd88 knockout mice. The primary bone marrow cells have been immortalized by infection with the ecotropic transforming replication-deficient retrovirus J2 and characterized for macrophage specific properties (BEI Resources, USA). These cell lines, including RAW264.7, were cultured in complete DMEM supplemented with Fetal Bovine Serum (10%) containing 1% broad-spectrum antibacterial and antifungal solutions. Standard tissue culture conditions (37°C and 5% CO2) were used to maintain these cell lines.

### 2.4 Estimation of cytokine secretion

A 6-well cell culture plate was used to seed 2 million cells/well of ΔTLR2, ΔTLR4, ΔTRIF, ΔMyd88, and RAW264 cells. Cells were maintained for 2 h at 37°C for adherence and infected with wild-type *Mycobacterium smegmatis* mc^2^155 (*Msmeg*), *Msmeg_VC* (vector control), *Msmeg_PE18*, *Msmeg_PPE26*, and *Msmeg_PE18+PPE26* at an MOI of 1:10. Untreated cells, *Msmeg*, and *Msmeg_VC* were used as negative controls. At 24 and 48 h after infection, the culture supernatant was collected and stored at -80°C until use. TNFα, IL6, IL12, IL1β, and IFN-γ levels were quantified using a mouse ELISA kit (BioLegend, USA). The absorbance at 450 nm and 570 nm was measured using an ELISA plate reader to determine the cytokine levels.

### 2.5 Intracellular survival of wild-type and recombinant *Msmeg* within RAW264.7 cells

A 6-well culture plate was used for seeding 2 million RAW264.7 cells/well. The cells were allowed to adhere overnight. *Msmeg*, *Msmeg_VC*, *Msmeg_PE18*, *Msmeg_PPE26*, and *Msmeg_PE18+PPE26* strains were grown till log phase (OD600nm-0.6) in standard Middlebrook 7H9 (BD DIFCO™, USA) medium supplemented with oleic acid-albumin-dextrose-catalase (OADC, BD DIFCO™, USA) and 0.1% Tween-80. The cells were collected and washed thrice with 1× cold PBS. The cells were then passed through a 26-gauge syringe to prepare a single-cell suspension. An MOI of 1:10 (~20 million bacteria/well) was used to infect the macrophages for 1 h. The infection rate was calculated to be approximately 12% using the formula: Infection Rate = (Average number of CFU after 1 h of infection/2 million cells) × 100. After infection, the cell culture media were removed, and infected cells were washed three times with 1× cold PBS followed by suspension in incomplete RPMI supplemented with gentamicin (Sigma) (10µg/ml) for 1 h to limit the growth of extracellular bacteria. This media was again replaced by complete RPMI and infection experiments were performed for 24–96 h. Wild-type and recombinant bacteria-infected RAW264.7 cells were lysed in sterile PBS containing Triton X-100 (1%). After lysis, bacterial cells were plated on 7H11 agar plates. The intracellular survival of wild-type and recombinant *Msmeg* was determined using the colony-forming unit (CFU) assay. On the fourth day of plating, growth was observed and the colonies were counted.

### 2.6 Western blot analysis of protein samples

6-well culture plate was used for culturing RAW264.7 cells (3 million cells/well). Cells were infected with *Msmeg*, *Msmeg_VC*, *Msmeg_PE18*, *Msmeg_PPE26*, or *Msmeg_PE18+PPE26* at an MOI of 1:10 for 24 h. Untreated cells, *Msmeg*, and *Msmeg_VC* served as the controls. The cells were harvested 24 h post-infection in sample loading buffer containing 100 mM dithiothreitol. Protein samples were prepared by heating at 95°C for 10 min, kept at 4°C for 10 min, and centrifuged at 13000 rpm for 5 min to remove cell debris. Protein samples were loaded on SDS-PAGE, followed by the transfer of proteins onto the PVDF membrane. PVDF membranes were blocked using a buffer containing skim milk or BSA (11, 42). Antibodies against Caveolin, EEA1, Rab11, Rab5, Rab7, Arp2, N-Wasp, Rac1, and Profilin were purchased from Cell Signaling Technology (USA). anti-GroEL2 (BEI Resources, USA), anti-GST (BioLegend, USA), and anti-GAPDH (Santa Cruz Biotechnology, USA) antibodies were used for the western blot analysis. αPE18, αPPE25, αPPE26, and αPPE27 antibodies were used to identify the subcellular localization of PE/PPE proteins in various *Mtb* fractions. Horseradish peroxidase-conjugated secondary antibodies were used for signal generation. Images were captured using a ChemiDoc imaging system (Bio-Rad Laboratories). Densitometric quantification of the protein bands was performed using ImageJ software. GAPDH was used for the normalization of the protein bands.

### 2.7 Ethics statement

The guidelines established by the CPCSEA, IBSC, and IAEC of the ICMR National Institute of Pathology (NIOP), New Delhi, were followed. All experiments involving animals were conducted according to prescribed principles (IBSC Code No. NIP/IBSC/2020 (1)/02). The in-house animal facility for NIOP is equipped with positive-pressure devices and maintains ambient temperature and light conditions (25°C, 12 h light/dark cycle). The IAEC approved 16 inbred C57BL/6 female mice for use in the experiments.

The IAEC of NIOP was also approved (Code No. 1801) four New Zealand female rabbits to produce polyclonal antibodies against the protein of interest. The Central Animal Facility of the NIOP was used to maintain the animals, as approved by the IAEC. The rabbits were returned to AIIMS New Delhi after completion of the experimental rehabilitation procedures.

### 2.8 Generation of anti-PE18, anti-PPE25, anti-PPE26, and anti-PPE27 antibodies in rabbit

Purified proteins PE18, PPE25, PPE26, and PPE27 (500 μg) were mixed with Freund’s incomplete adjuvant and injected into rabbits at four different sites (125 µL/site). To generate polyclonal antibodies in rabbits, 200–800 µg of antigen is recommended for the first injection, followed by half the concentration in consecutive booster doses (45, 46). Two booster doses were administered 21 days after primary immunization (250 μg protein for each antigen) with Freund’s incomplete adjuvant. Western blot analysis was used to determine the specificity of polyclonal antibodies, as shown (**Supplementary Figure S2B**).

### 2.9 Quantitation of nitric oxide secreted by RAW264.7 cells after *Msmeg* stimulation

A 24-well culture plate was used to seed 1 million RAW264.7 cells/well. After adherence, the cells were infected with *Msmeg*, *Msmeg_VC*, *Msmeg_PE18*, *Msmeg_PPE26*, or *Msmeg_PE18+PPE26* at an MOI of 1:10 for 4 h at 37°C. The cells were washed thrice with 1× cold PBS. The cells were incubated for 1 h in complete DMEM containing gentamicin to kill extracellular bacteria. At 24 and 48 h post-infection, the culture supernatant was collected, and NO levels were measured using Griess reagent.

#### 2.9.1 Pull-down assay

Competent BL21 (DE3) cells were transformed with a combination of clones (1) pET-28a_*ppe26* and pGEX-6P2_*pe18* (2); pET-28a_*ppe25* and pGEX-6P2_*pe19* (20); pET-28a_*ppe27* and pGEX-6P2_*pe18*; (4) pET-28a_*ppe25* and pGEX-6P2_*espg5*; (5) pET-28a_*ppe26* and pGEX-6P2_*espg5*; and (6) pET-28a_ *ppe27* and pGEX-6P2_ *espg5*. Positive clones were selected on kanamycin (50 μg/ml)-and ampicillin (100 μg/ml)-containing agar plates and incubated at 37°C for 16 h. Cells from positive clones were grown overnight in LB broth as the primary culture. The secondary culture was started and grown for 4 h until the OD600nm reached 0.6. Recombinant protein production was induced using 0.5 mM IPTG (Isopropyl β-D-1-thiogalactopyranoside). Cultures were then pelleted and resuspended in 25 ml of lysis buffer (11, 42). Cells were lysed by sonication [three cycles (10 s ON and 60 s OFF) at 30% amplitude)]. The lysed cells were centrifuged at 13000 rpm for 45 min. The Ni-NTA affinity column was loaded with the supernatant to bind the protein. The column was washed with lysis buffer containing 20 mM imidazole (11, 42). The protein complexes were eluted in elution buffer containing 200 mM imidazole (11, 42). Protein samples were prepared by heating at 95°C in sample loading buffer. Western blot analysis was performed using αHis (Sigma, USA) and αGST antibodies (BioLegend, USA).

#### 2.9.2 Immunization of mice

Inbred C57BL/6 mice were randomized into four groups (*n* = 4/group). Mice were immunized with PE18 (group 1), PPE26 (group 2), PE18+PPE26 (group 3), or PBS control (group 4). Primary immunization was performed with purified PE18, PPE26, and PE18+PPE26 proteins (30 μg) in 100 μl 1× PBS (pH 7.4), as suggested for the protein dose in mouse immunization (47, 48).Two booster doses of proteins were administered after the primary immunization at 15 days intervals (15 µg of protein in 100 μl PBS). The mice in the control group received an equivalent volume of 1× PBS. After immunization, mice were euthanized.

#### 2.9.3 Harvesting of splenocytes and peritoneal macrophages

For the isolation of peritoneal macrophages, 5 ml chilled 1× PBS was injected into the peritoneum. PBS along with the cells was collected using a syringe. The spleen was isolated from individual mice, stored in chilled PBS, and gently crushed and perfused using a 26-gauge syringe needle. A cell strainer was used to remove cell debris from the suspension. The cell suspension was centrifuged and mixed in RBC lysis buffer (Thermo Fisher Scientific, USA). After centrifugation, the splenocytes devoid of erythrocytes were collected in complete RPMI media.

#### 2.9.4 Staining of peritoneal macrophages for analyzing the expression of activation markers

Peritoneal macrophages from primed mice were isolated and cultured in 24 well plates (0.5 million cells/well). Macrophages were stimulated with PE18 (5 μg), PPE26 (5 μg), and PE18 (2.5 μg) + PPE26 (2.5 μg). The expression of macrophage surface markers (CD80, MHCI, and MHCII) was determined. Cells were harvested after 24 h of stimulation and incubated with FITC-A:F4/80, APC-A:CD80, APC-Cy7-A:MHCI, and PE-Cy7-A: MHCII antibodies (BioLegend, USA). 100 ng/ml LPS and 10 µg Concanavalin A (ConA) treated cells were used as positive controls. Sample readings were acquired using a flow cytometer (BD FACSAria) (BD Biosciences, USA). FlowJo™ 10 (BD Biosciences, USA) was used for analyzing the data.

#### 2.9.5 Surface marker staining of splenocytes

24 well plate was used for seeding splenocytes isolated from primed mice (0.1 million cells/well). The PE18, PPE26, and PE18+PPE26 proteins were used to stimulate splenocytes for 48 h. The cells were washed with FACS buffer (1× PBS + 2% FBS). Anti-mouse APC-Cy7-A:CD3, PerCP-Cy5.5-A:CD4, and FITC-A:CD8 antibodies were used to stain surface markers. Formaldehyde (4%) was used to fix cells. The memory response was assessed after stimulation of splenocytes with PE18, PPE26, and PE18+PPE26 proteins using fluorophore-tagged antibodies (PE-A:CD44 and APC-A:CD62L). Ten thousand events were acquired using a FACSAria flow cytometer. FlowJo™ 10 (BD Biosciences, USA) was used for the analysis of data.

#### 2.9.6 Statistical analysis

Data analysis was performed using one-way or two-way ANOVA with appropriate *post-hoc* tests, as required and mentioned in the respective figure legends. GraphPad Prism 9 was used for data analysis (11).

## 3 Results

### 3.1 ESX-5 encoded PE and PPE proteins interacted with each other to perform coordinated functions

The *pe/ppe* genes of the ESX-5 genomic locus were organized into a single continuous stretch of DNA. The ESX-5 encoded proteins are expected to functionally interact with each other. STRING protein-protein interaction network analysis was used to reveal the interaction between PE and PPE proteins and the chaperone EspG5. STRING data analysis showed interactions between PE18-PPE26-EspG5, PE19-PPE25-EspG5 and PE18-PPE27-EspG5, (**Supplementary Figures S3A–C**). To confirm the predicted interactions, we performed molecular docking analysis of PE18 and PPE26, PE19 and PPE25 and PE18 and PPE27. The high docking score and extensive hydrogen-bond networks suggested a physical interaction between these PE/PPE proteins (**Supplementary Figures S3A–C**, **Supplementary Table S2**). The cognate chaperone EspG5 was also docked to PPE proteins PPE26, PPE25, and PPE27. The docking results suggested an interaction between EspG5 and PPE proteins PPE26, PPE25, and PPE27 (**Supplementary Figures S3A–C**, **Supplementary Table S2**). STRING protein interaction network analysis and molecular docking results suggest that the ESX-5 encoded PE/PPE proteins may interact with each other, and the chaperone EspG5 interacts with the PE/PPE heterodimeric complexes for efficient transport across the *Mtb* cell envelope.

**Figure 1:**
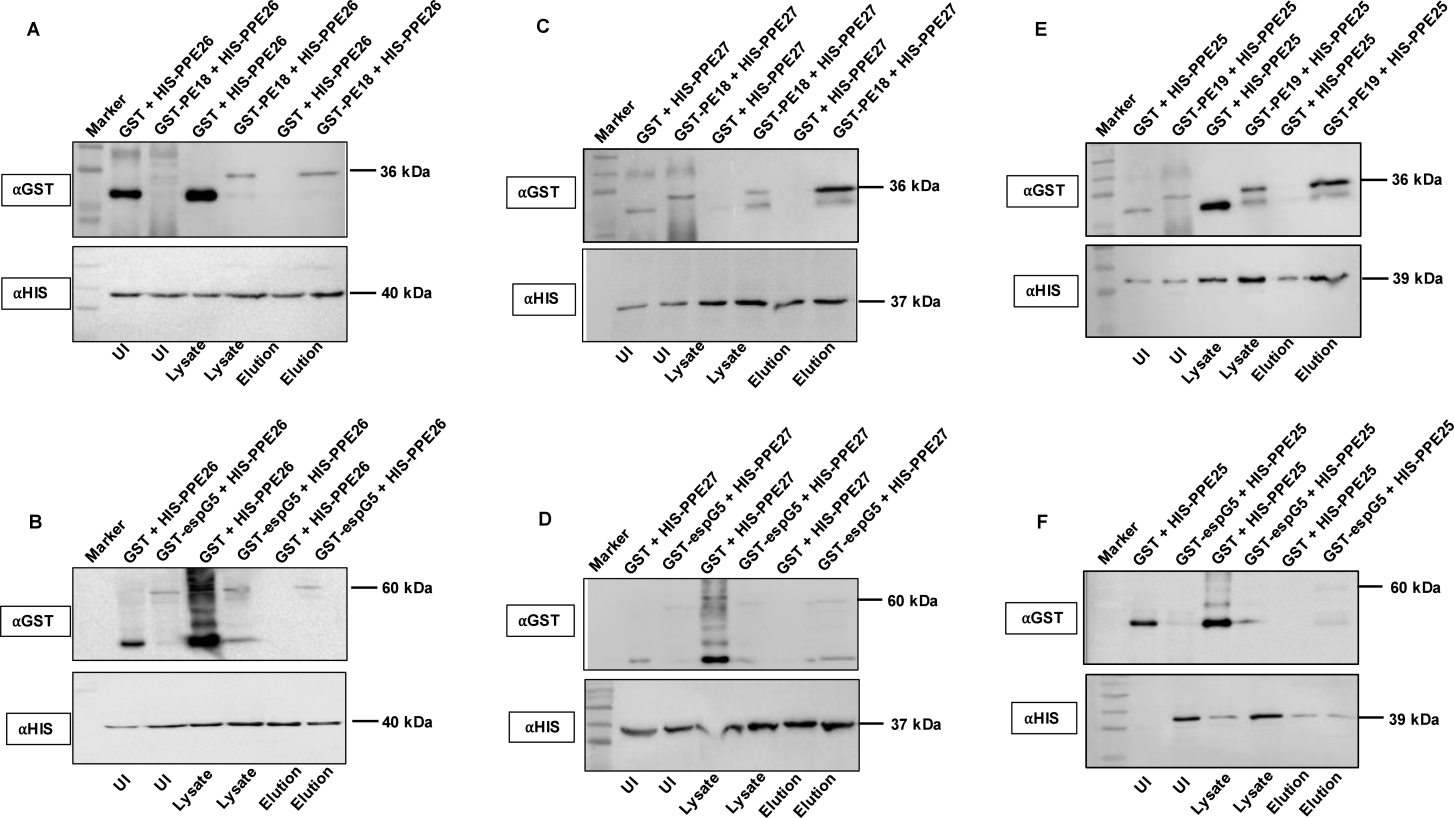
PE18 and PPE26 proteins form heterodimeric complex, and EspG5 interacts with PPE25, PPE26, and PPE27. To study the interaction between PE and PPE proteins of ESX-5 and the chaperone EspG5, we co-expressed PE18+PPE26, PPE26+EspG5, PE19+PPE25, PPE25+EspG5, PE18+PPE27, and PPE27+EspG5 as GST and His-tagged proteins. Co-expressed protein lysates were used in the His-pull-down assay to determine direct interactions between these proteins. Protein lysates from cells co-expressing GST and His-tagged PPE26, PPE25, and PPE27 were used as negative controls. **(A, C, E)** Western blotting showing the co-expression profile and interaction of PE18 with PPE26, PE18 with PPE27, and PE19 with PPE25. **(B, D, F)** Western blot analysis showing the interactions between PPE26 and EspG5, PPE27 and EspG5, and PPE25 and EspG5. The sizes of the protein bands and the antibodies used are marked in the figure. Three independent experiments were performed to validate the protein-protein interactions.

To strengthen our *in silico* findings, we performed a His pull-down assay on bacterial cell extracts prepared from co-expressed His and GST-tagged PE18 and PPE26, PE18 and PPE27, PE19 and PPE27, and His-tagged PPE26, PPE27, and PPE25 with GST-tagged EspG5. The pull-down assay showed that PE18 interacted with PPE26 and PPE27, and PE19 interacted with PPE27 (**Figures 1A, C, E**). The results revealed that the ESX-5 encoded chaperone EspG5 interacted with PPE26, PPE27, and PPE25 (**Figures 1B, D, F**). Soluble protein lysates from GST + His-tagged co-expressed PE/PPE proteins were used as negative controls (**Figures 1A–F**). Together, these observations suggest that ESX-5 encoded PE18 interacts with PPE26 and PPE27, whereas PE19 forms a heterodimeric complex with PPE27. The resultant heterodimeric complexes may be transported to the *Mtb* cell envelope by ESX-5 T7SS to execute their cooperative functions. We also explored the homodimeric interactions between PE proteins of ESX-5, PE18, and PE19. His and GST-tagged PE18 and PE19 were co-expressed in *E. coli*, soluble protein lysates were prepared, and a His pull-down assay was performed. His-tagged PE18 and PE19 efficiently precipitated the GST-tagged PE18 and PE19 proteins (**Supplementary Figures S4A, B**). These results suggest that the ESX-5 encoded PE18 and PE19 form a homodimeric complex in *Mtb*. Heterodimeric complex formation has been reported for the PE proteins PE9 and PE10 (49). Circular dichroism spectroscopy was used to study the secondary structure of the purified protein before performing protein-protein interactions (**Supplementary Figures S4C–E**).

### 3.2 Subcellular localization of PE and PPE proteins (PE18, PP25, PPE26, and PPE27) in *Mtb*

**Figure 2:**
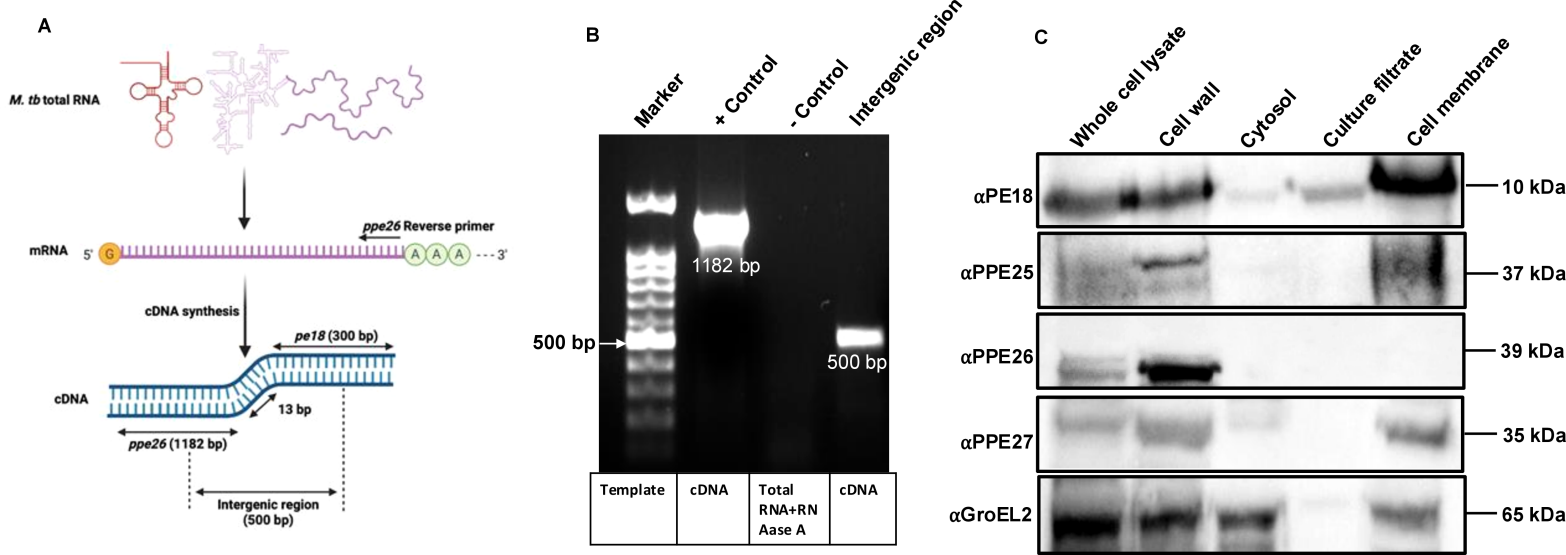
Surface localized proteins of *pe18* and *ppe26* genes constitute an operon in *Mtb.* **(A)** Pictorial representation of total RNA and the scheme adopted for amplifying *pe18* and *ppe26* using cDNA prepared from total RNA. Primers used to amplify *the ppe26* entire region and parts of the genes (*pe18* and *ppe26*), including the intergenic region, are marked in the figure. Two independent experiments were performed to show the operonic nature of *pe18* and *ppe26* genes. **(B)** Agarose gel showing the amplified products visualized by ethidium bromide staining. Total RNA treated with DNase I and RNase A was used as the control. Bands containing *ppe26* and regions of *pe18* and *ppe26*, including the intergenic regions, are shown in the figure. **(C)** Subcellular localization of PE18, PPE25, PPE26, and PPE27 in various fractions of *Mtb*, including total protein or whole cell lysate, enriched cell wall fraction, cytosol, secretory fraction, and total membrane. 20µg of each fraction were used for western blot analysis using αPE18, αPPE25, αPPE26, and αPPE27 antibodies (1:1000). The antibodies used and the molecular weights of the proteins are shown in the figure. GroEL2 localization in *Mtb* was used as the control.

Functional localization is related to the transcriptional organization of genes. Bacterial genes that are organized in operons functionally interact with each other. The interaction between PE18 and PPE26 prompted us to identify whether these genes are co-operonic in *Mtb*. Total RNA was isolated from *Mtb* H37Rv cell lysates, cDNA was synthesized, and PCR was performed. An entire region containing PPE26 was amplified using gene-specific primers (**Figures 2A, B**, **Supplementary Table S1**). We also used primers that recognize the intergenic regions of PE18 and PPE26, as shown in **Figure 2A** and **Supplementary Table S1**. The PCR-amplified products were fractionated on an agarose gel and stained with ethidium bromide. Successful amplification was observed that contained the PPE26 entire region (1182 bp) and the PE18 and PPE26 intergenic region (500 bp) (**Figure 2B**). RNA digested with RNase A and DNase I were used as the negative and positive controls, respectively. These findings demonstrate that PE18 and PPE26 constitute an operon, suggesting that these proteins may be functionally related or perform similar functions in host-pathogen interactions, *Mtb* physiology, and immune modulation. To confirm the cellular localization of PE and PPE proteins of ESX-5, we used purified cellular fractions of *Mtb* in western blot analysis with αPE18, αPPE25, αPPE26, and αPPE27 polyclonal antibodies (generated in-house). We found that PPE family protein PPE26 was only localized in the cell wall fraction. PE18 was mainly found in the cell wall, culture filtrate, and total membrane fractions, whereas PPE25 and PPE27 were localized in the cell wall and cell membrane fractions (**Figure 2C**). The localization of GroEL2 was used as a control (**Figure 2C**). These results suggest that PE18, PPE26, and PPE25 are localized at the host-pathogen interface and may be involved in immune modulation.

### 3.3 PE18, PPE26, and PE18+PPE26 of *Mtb* elicit the production of proinflammatory cytokines

The observation that PPE26 is a cell wall-associated protein and that PE18 is a cell wall and secretory protein of *Mtb* suggests that these proteins may regulate host-pathogen interactions during infection. Therefore, to confirm their role in immune modulation, we used recombinant *Msmeg* expressing PE18, PPE26, and PE18+PPE26 to infect RAW264.7, ΔTLR2, ΔTLR4, and ΔTRIF cells (MOI, 1:10). *Msmeg* harbors only ESX-1, ESX-3, and ESX-4 T7SS lacking ESX-5, whereas *Mtb* contains five ESX T7SS (ESX-1 to ESX-5) (50, 51). Moreover, it has been shown that PE/PPE proteins of *Mtb* expressed in *Msmeg* are targeted to the cell wall and are surface-exposed (38, 52–55). Furthermore, *Msmeg* is a valuable surrogate for studying *M. tb* genes due to its non-pathogenicity, rapid growth, and genetic homology, including conserved gene orthologs and similar physiology. It facilitates functional analysis of *Mtb* genes, avoiding biosafety constraints and prolonged generation times. *Msmeg* has been pivotal in elucidating the mechanisms of TB drugs like isoniazid and ethambutol (56).

**Figure 3:**
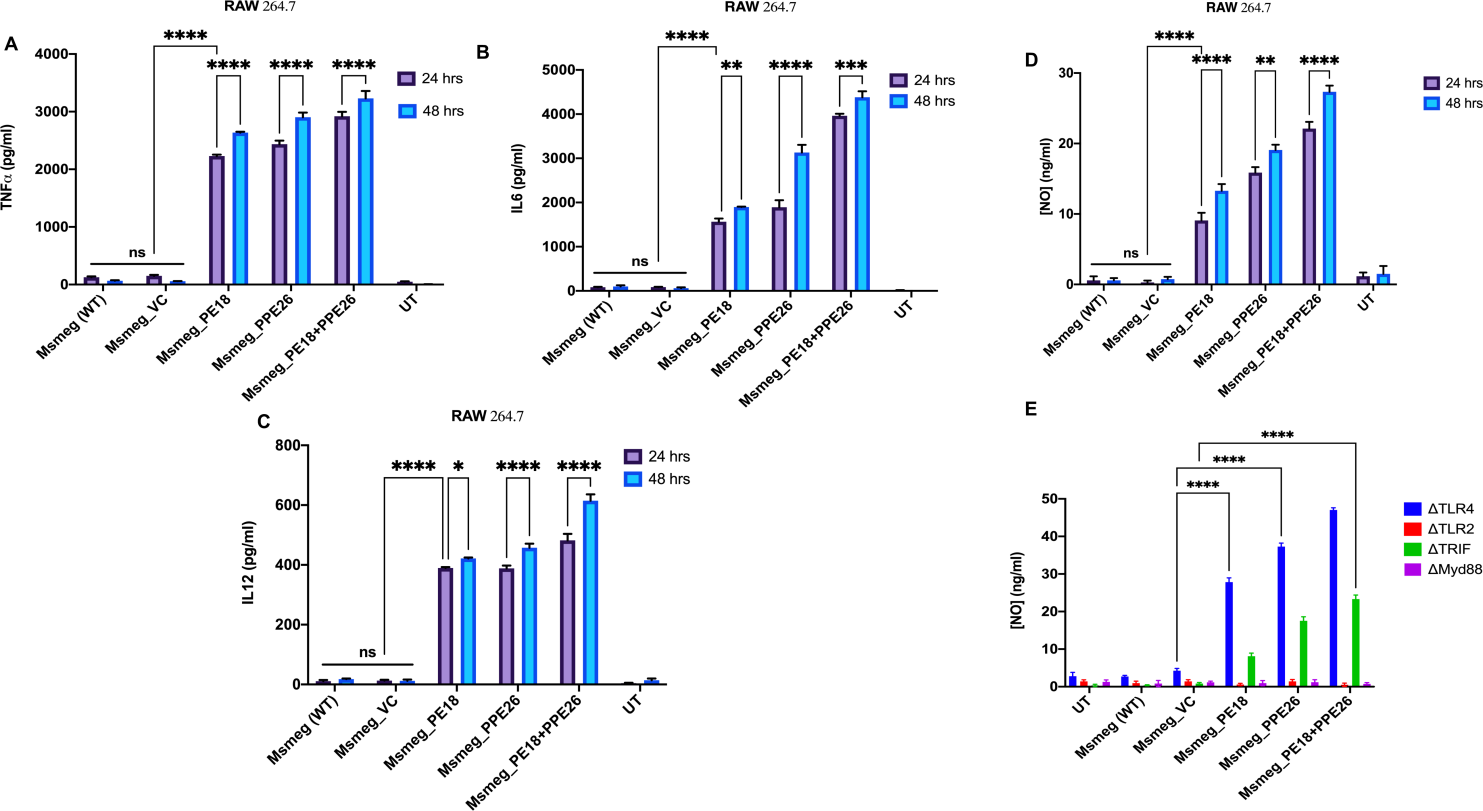
The secretion of proinflammatory cytokines and NO are induced by PE18 and PPE26 proteins in macrophages. **(A–C)** A 6-well tissue culture plate was used for seeding 2 million RAW264.7 macrophage cells. The cells were incubated overnight for adherence. After adherence, cells were infected with *Msmeg*, *Msmeg_VC*, and *Msmeg* expressing PE18, PPE26, and PE18+PPE26 (MOI = 1:10). Culture supernatants were collected after 24 and 48 h of infection. The levels of TNFα, IL6, and IL12 were quantified using ELISA. **(D, E)** RAW264.7, mouse macrophages were infected with *Msmeg*, *Msmeg_VC*, and *Msmeg* expressing PE18, PPE26, and PE18+PPE26 for 24 h. The culture supernatant was collected, and NO levels were quantified using Griess reagent. Untreated cells (UT), *Msmeg* (wild-type), and *Msmeg_ VC-infected* RAW264.7 cells were used as controls. Three independent experiments were performed to determine the level of pro-inflammatory cytokines and NO. Two-way ANOVA was used to analyze the data. Tukey’s multiple comparisons test was applied post-test. *P values less than 0.05, **P values less than 0.01, ***P values less than 0.001, ****P values less than 0.0001 as compared to the controls. n. s shows that the data were not significant.

**Figure 4:**
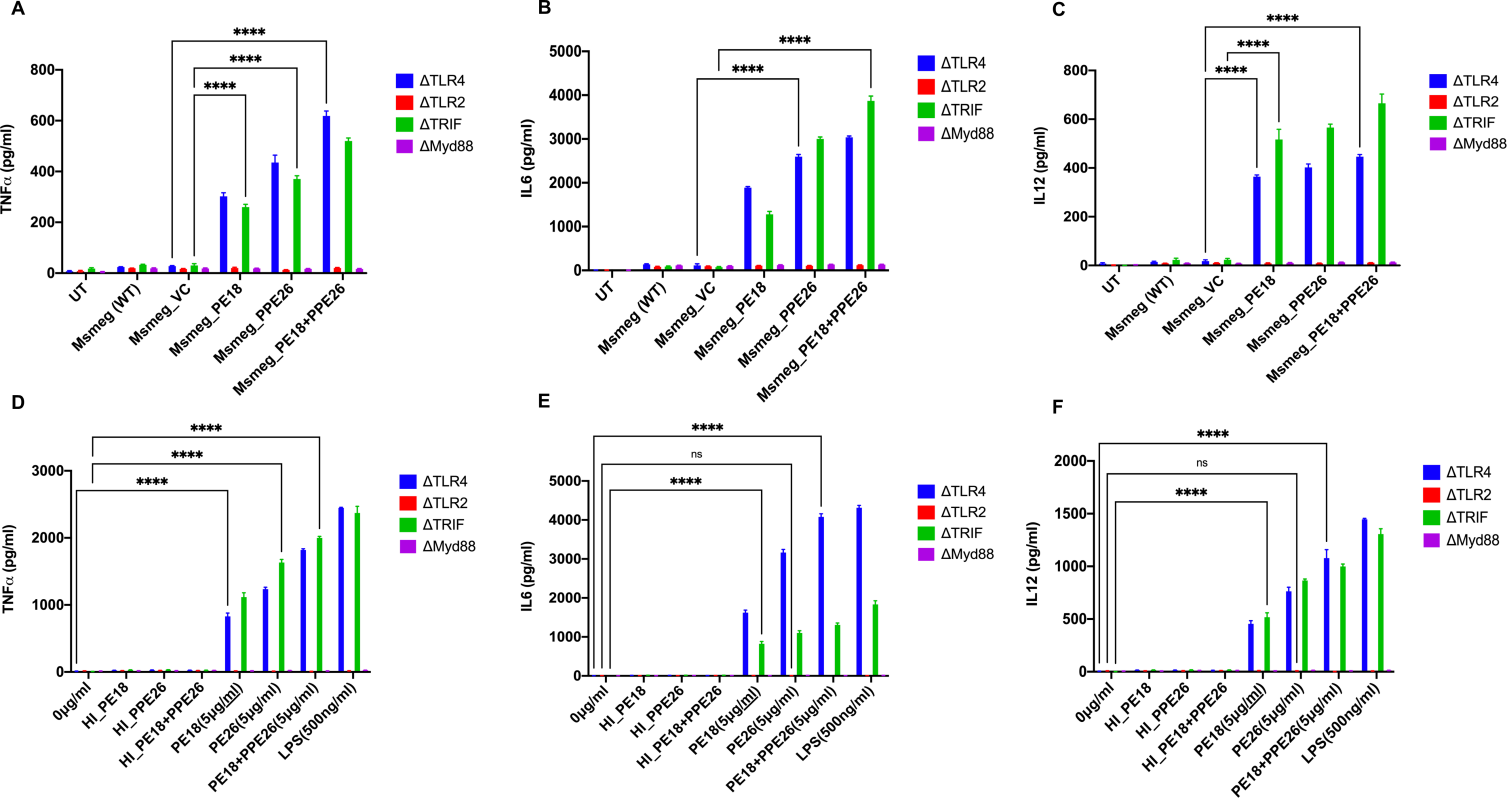
The innate immune receptor TLR2 and adaptor Myd88 are involved in the secretion of proinflammatory cytokines. **(A-C)** 6-well tissue culture plates were used for seeding 2 million TLR knockout macrophage cells. The cells were incubated overnight for adherence. After adherence, cells were infected with *Msmeg*, *Msmeg_VC*, and *Msmeg* expressing PE18, PPE26, and PE18+PPE26 (MOI = 1:10). Culture supernatants were collected after 24 and 48 h of infection. The levels of TNFα, IL6, and IL12 were quantified using ELISA. **(D-F)** 6-well culture plates were used for seeding ΔTLR2, ΔTLR4, ΔTRIF, and ΔMyd88 mouse macrophages (2 million cells/well) and allowed to adhere for 2 h. PE18 (5 µg), PPE26 (5 µg), and PE18 (2. 5µg) + PPE26 (2.5 µg) purified proteins were used to treat macrophage cells for 24 h. The culture supernatant was collected, and TNFα, IL6, and IL12 levels were measured using sandwich ELISA. Untreated (UT) cells, Proteinase K-digested, and heat-inactivated protein (HI) (5 µg)-treated cells were used as negative controls. Three independent experiments were performed to determine the level of pro-inflammatory cytokines. ****P values less than 0.0001 as compared to the controls. n. s shows that the data were not significant.

The secretion of cytokines (proinflammatory and anti-inflammatory) was analyzed at 24 and 48 h post-infection. Recombinant *Msmeg* containing *PE18, PPE26*, and *PE18+PPE26* infected macrophages (RAW264.7) exhibited increased production of TNFα, IL6, and IL12 (**Figures 3A–C**), with a similar increase observed in ΔTLR4 and ΔTRIF macrophages (**Figures 4A–C**). In contrast, ΔTLR2 and ΔMyd88 cells did not release these cytokines (**Figures 4A–C**). To further confirm the role of PE18 and PPE26 in the production of proinflammatory cytokines, we used the purified proteins PE18, PPE26, and PE18+PPE26 to treat ΔTLR2, ΔTLR4, ΔMyd88, and ΔTRIF cells. After 24 h of protein treatment, cytokine levels were estimated using ELISA. PE18, PPE26, and the combination of the two protein treatments induced enhanced secretion of TNFα, IL6, and IL12 in the order PE18<PPE26<PE18+PPE26, with HI used as the heat-inactivated negative control which demonstrated that the purified proteins were devoid of endotoxin contamination (**Figures 4D–F**). Intriguingly, we observed that ΔTLR4 and ΔTRIF macrophages secreted significant amounts of cytokines (**Figures 4D–F**). Similar to the infection studies, there was negligible secretion of proinflammatory cytokines by ΔTLR2 and ΔMyd88 cells, confirming that PE18 and PPE26 elicit production of proinflammatory cytokines by murine macrophages mediated by the innate immune receptor TLR2 and adapter Myd88 (**Figures 4D–F**).

Moreover, *Msmeg* expressing PE18, PPE26, and PE18+PPE26 induced high NO production by RAW264.7 and ΔTRIF cells, whereas no such release of NO was observed in ΔTLR2 and ΔMyd88 cells (**Figures 3D, E**). Intriguingly, we observed maximum induction of NO and pro-inflammatory cytokine secretion by RAW264.7, ΔTLR4, and ΔTRIF cells infected with recombinant *Msmeg* expressing PE18+PPE26, suggesting their synergistic function (**Figures 3A–E**, **Figures 4A–C**). These results demonstrate that PE18 and PPE26 elicit the production of proinflammatory cytokines and NO, mediated by the innate immune receptor TLR2 and adapter Myd88.

### 3.4 PE18 and PPE26 primed splenocytes exhibited Th1 polarization with an enhanced effector memory phenotype

**Figure 5:**
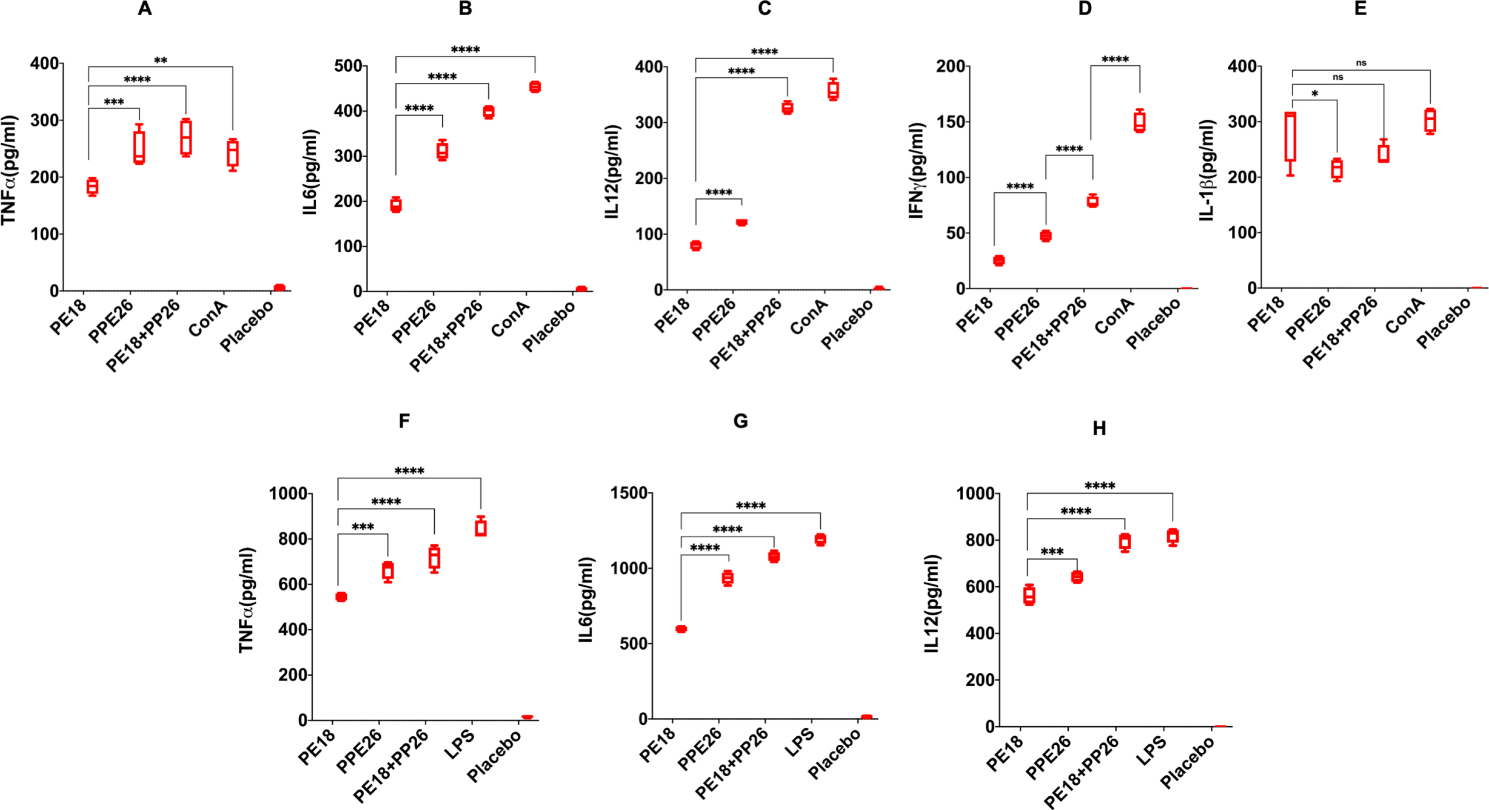
Proinflammatory cytokine production by splenocytes and peritoneal macrophages are induced by PE18 and PPE26 proteins. Splenocytes and peritoneal macrophages were isolated from PE18-, PPE26-, and PE18+PPE26 immunized mice. Splenocytes and peritoneal macrophages harvested from PBS-treated mice were used as the controls. Isolated splenocytes and peritoneal macrophages were cultured in complete RPMI and DMEM. After 2 h of culturing, cells were stimulated with PE18 (5 µg), PPE26 (5 µg), PE18 (2.5 µg) + PPE26 (2.5 µg) for 24 h. The culture supernatant was collected, and ELISA was used to measure cytokine levels. ConA (10 µg) and LPS (100 ng/ml) treatment of splenocytes and peritoneal macrophages was used as controls to produce proinflammatory cytokines. **(A–E)** After stimulation of splenocytes, the levels of TNFα, IL6, IL12, IFNγ, and IL1β were measured and are shown using scattered plots. **(F-H)** Scatter plots showing the levels of TNFα, IL6, and IL12 secreted by protein-restimulated peritoneal macrophages. Three independent experiments were performed to determine the level of pro-inflammatory cytokines secreted by peritoneal macrophages and splenocytes. Two-way ANOVA was used to analyze the data. Tukey’s multiple comparisons test was applied post-test. *P values less than 0.05, **P values less than 0.01, ***P values less than 0.001 and, ****P values less than 0.0001 compared to controls. n. s shows that the data were not significant.

Different groups of mice were immunized with PE18, PPE26, and PE18+PPE26 proteins, whereas PBS-treated mice were used as controls. Splenocytes and peritoneal macrophages from immunized mice were harvested, cultured, and treated with endotoxin-free purified PE18, PPE26, or PE18+PPE26. Untreated splenocytes and macrophages were used as negative controls. ConA and LPS treatments of splenocytes and macrophages were used as positive controls (Con A in **Figures 5A–E**, and LPS in **Figures 5F–H**). Priming splenocytes *in vitro* with PE18, PPE26, and PE18+PPE26 resulted in increased levels of TNFα, IL6, IL12, IFNγ, and IL1β (**Figures 5A–E**). Interestingly, we observed that PE18, PPE26, and PE18+PPE26 enhanced the secretion of TNFα, IL6, and IL12 by peritoneal macrophages (**Figures 5F–H**). The maximum secretion of proinflammatory cytokines was observed when PE18 and PPE26 were used together to treat splenocytes and macrophages (**Figures 5A–H**). These results strongly suggest that the PE/PPE proteins PE18 and PPE26 are proinflammatory antigens of *Mtb*, triggering the secretion of cytokines from primary splenocytes and macrophages. Interestingly, PE18 and PPE26 showed synergistic effects, suggesting that these PE/PPE family proteins function together to modulate the host immune functions.

**Figure 6:**
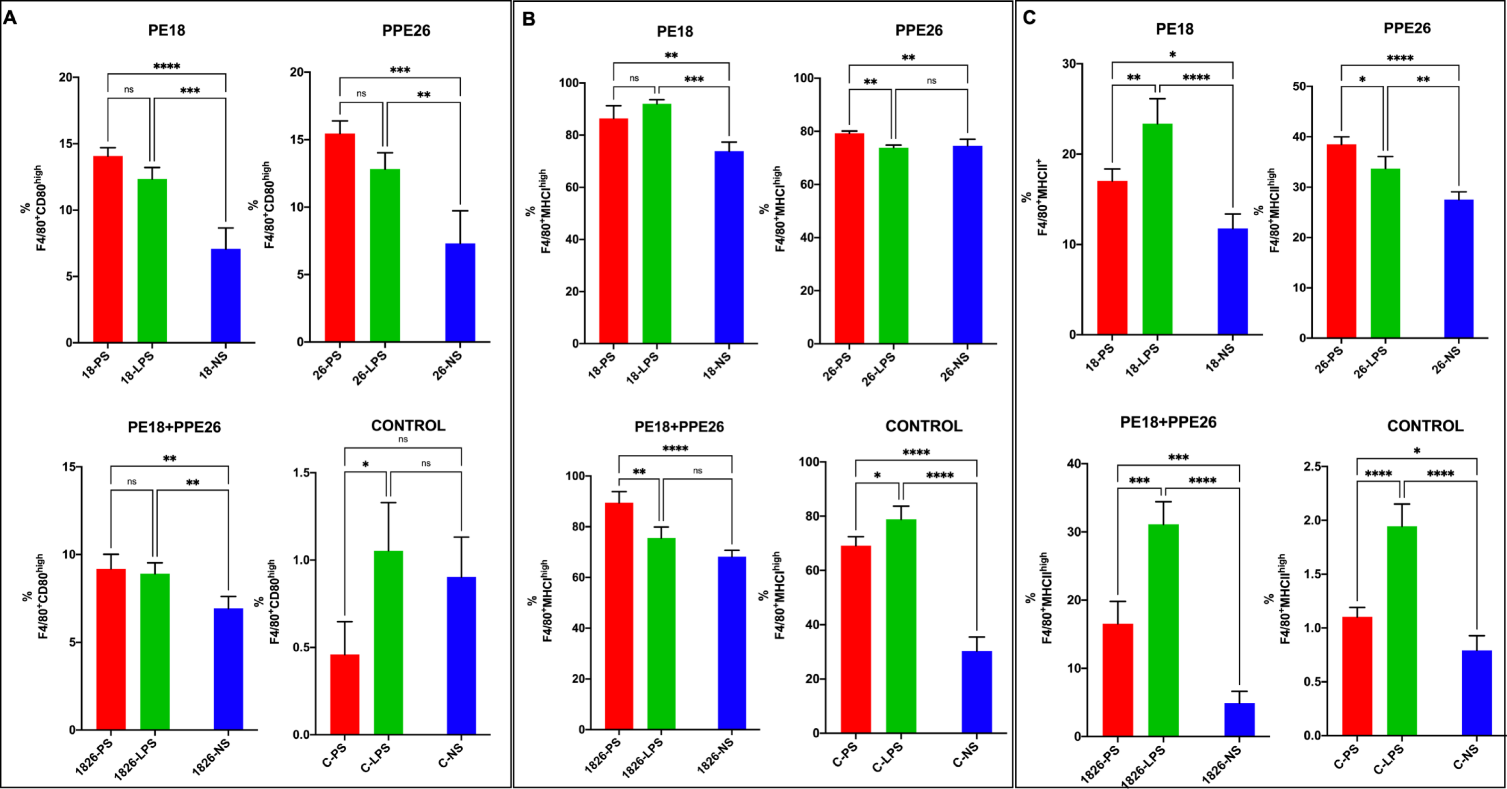
The production of macrophage activation markers is evoked by PE18 and PPE26 of *Mtb.* After the primary immunization of mice, two booster doses were administered with PE18, PPE26, and PE18+PPE26 proteins. PBS-treated mice were used as controls. Protein- and PBS-treated mice were sacrificed. Peritoneal macrophages were isolated and cultured in complete DMEM. The cells were allowed to adhere for 2 h. Post-adherence cells were stimulated with PE18 (5 µg), PPE26 (5 µg), PE18 (2.5 µg) + PPE26 (2.5 µg) for 24 h. After stimulating macrophages with the desired proteins, the cells were collected and stained with fluorophore-tagged αCD80, αMHCI, and αMHCII antibodies. Sample readings were acquired using a BD FACSAria flow cytometer. Data analysis was performed using FlowJo™10. UT- and LPS-treated cells were used as the controls. **(A)** Expression of CD80 costimulatory molecules on the macrophage surface is shown as a bar graph. **(B, C)** Expression levels of MHCI and MHC II on macrophage surfaces are shown as bar graphs. The names of the proteins used in the treatments are shown in the figure. Three independent experiments were performed to determine the level of macrophage activation markers. Two-way ANOVA was used to analyze the data. Tukey’s multiple comparisons test was applied post-test. *P values less than 0.05, **P values less than 0.01, ***P values less than 0.001 and, ****P values less than 0.0001 as compared to controls. n. s shows that the data were not significant. NS (non-restimulated), PS (protein restimulated).

To further validate their role in immune function regulation, peritoneal macrophages were isolated, cultured, and re-stimulated with PBS (control), PE18 (5 µg), PPE26 (5 µg), and PE18 (2.5 µg) + PPE26 (2.5 µg). The cells were harvested 24 h after restimulation and labeled with anti-CD80, anti-MHC1, and anti-MHCII antibodies tagged with fluorophores, followed by multi-parameter FACS analysis. We found that secondary PE18, PPE26, and PE18+PPE26 protein exposure to peritoneal macrophages isolated from the respective protein-immunized mice showed increased expression of the costimulatory molecule CD80 and activation markers MHCI and MHCII in peritoneal macrophages isolated from the immunized mice compared to the PBS-treated mice (**Figures 6A–C**, **Supplementary Figures S5**–**S7**). While PPE26 and PE18+PPE26 exposure resulted in significantly higher activation of MHCI compared to the LPS re-stimulated and PBS re-stimulated control, PE18 showed non-significant MHCI upregulation compared to LPS re-stimulation and significant upregulation compared to PBS re-stimulated control. Further, PPE26 re-stimulation showed similar higher MHCII activation to that of LPS re-stimulated control as compared to the non-restimulated control group. These results exclusively indicate that *Mtb* proteins PE18 and PPE26 activate macrophages to perform immunomodulatory functions during host-pathogen interactions.

**Figure 7:**
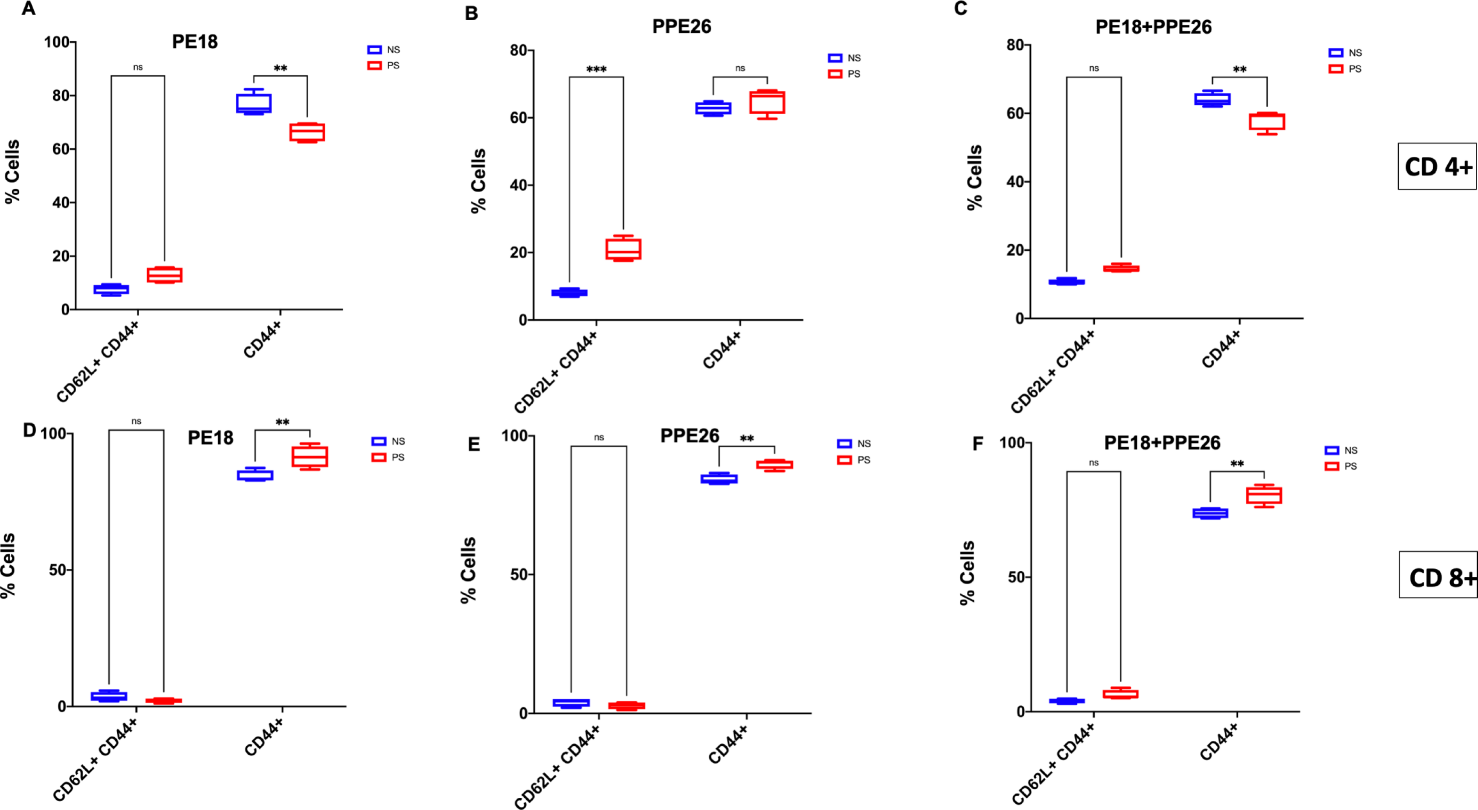
PE18 and PPE26 proteins of *Mtb* regulate T-cell memory response. After primary immunization of mice, two booster doses were administered with PE18, PPE26, and PE18+PPE26 proteins. PBS-treated mice were used as controls. Protein- and PBS-treated mice were sacrificed and splenocytes were isolated and cultured in complete RPMI medium. Cells were stimulated with PE18 (5 µg), PPE26 (5 µg), PE18 (2.5 µg) + PPE26 (2.5 µg) for 48 h. Stimulated splenocytes were harvested and stained with fluorophore tagged αCD3, αCD4, αCD8, αCD44, and αCD62L antibodies. Sample readings were acquired using a BDFACSAriall flow cytometer, and data were analyzed using FlowJo™10. Non-restimulated cells were used as the controls. **(A-C)** CD44 and CD62L memory marker expression in CD3+ and CD4+ T cells stimulated with PE18, PPE26, and PE18+PPE26, as shown in the bar graphs. **(D-F)** CD44 and CD62L memory marker expression in CD3+ and CD8+ T cells treated with PE18, PPE26, and PE18+PPE26, as shown in the bar graphs. Three independent experiments were performed to determine the expression of T cell memory markers. Two-way ANOVA was used to analyze the data. Tukey’s multiple comparisons test was applied post-test. **P values less than 0.01, ***P values less than 0.001 compared to controls. n. s show that the data were not significant.

Further, to confirm the roles of PE18 and PPE26 proteins in immune modulation for generating memory response, mouse splenocytes were cultured and re-stimulated with PE18 (5 µg), PPE26 (5 µg), and PE18 (2.5 µg) + PPE26 (2.5 µg). Stimulated cells were treated with fluorophore-tagged αCD3, αCD4, αCD8, αCD62L, and αCD44 antibodies, followed by FACS analysis. FACS results showed that PE18 and PPE26 enhanced CD62L+CD44+ memory marker expression in CD4+ T-cells compared to T-cells isolated from control mice (**Figures 7A–F**, **Supplementary Figure S8**). Notably, CD44 expression was higher in CD8+ T-cells following re-stimulation with PE18, PPE26, and PE18+PPE26, compared to the non-restimulated group. However, CD4+ T-cells in the PE18 and PE18+PPE26 re-stimulated groups showed a marked decrease in CD44 expression (with prominent decrease in the latter condition). This suggests that the activation of the Th1 cell population may lead to an enhanced response upon re-stimulation or re-infection, particularly in the groups with significantly higher activation levels (**Figures 7A–F**). PPE26 re-stimulation expressed a significantly higher CD44+CD62L+ population than the non-restimulated group in CD4+ cells, suggesting its role in the establishment of central memory response (**Figure 7B** **).** Interestingly, we also observed that PE18 and PPE26 skewed the CD4+/CD8+ ratio, which suggested an increased expansion of CD8+ T-cells after restimulation with the protein antigens (**Supplementary Figure S9**). Together, these results suggest that *Mtb* PE18 and PPE26 regulate memory marker expression in CD4+ and CD8+ T cells, suggesting that these PE/PPE family proteins play a crucial role in modulating adaptive immunity.

### 3.5 PE18 and PPE26 enhance intracellular survival by hampering the endosome-phagosome dysregulation due to alteration in actin assembly dynamics

**Figure 8:**
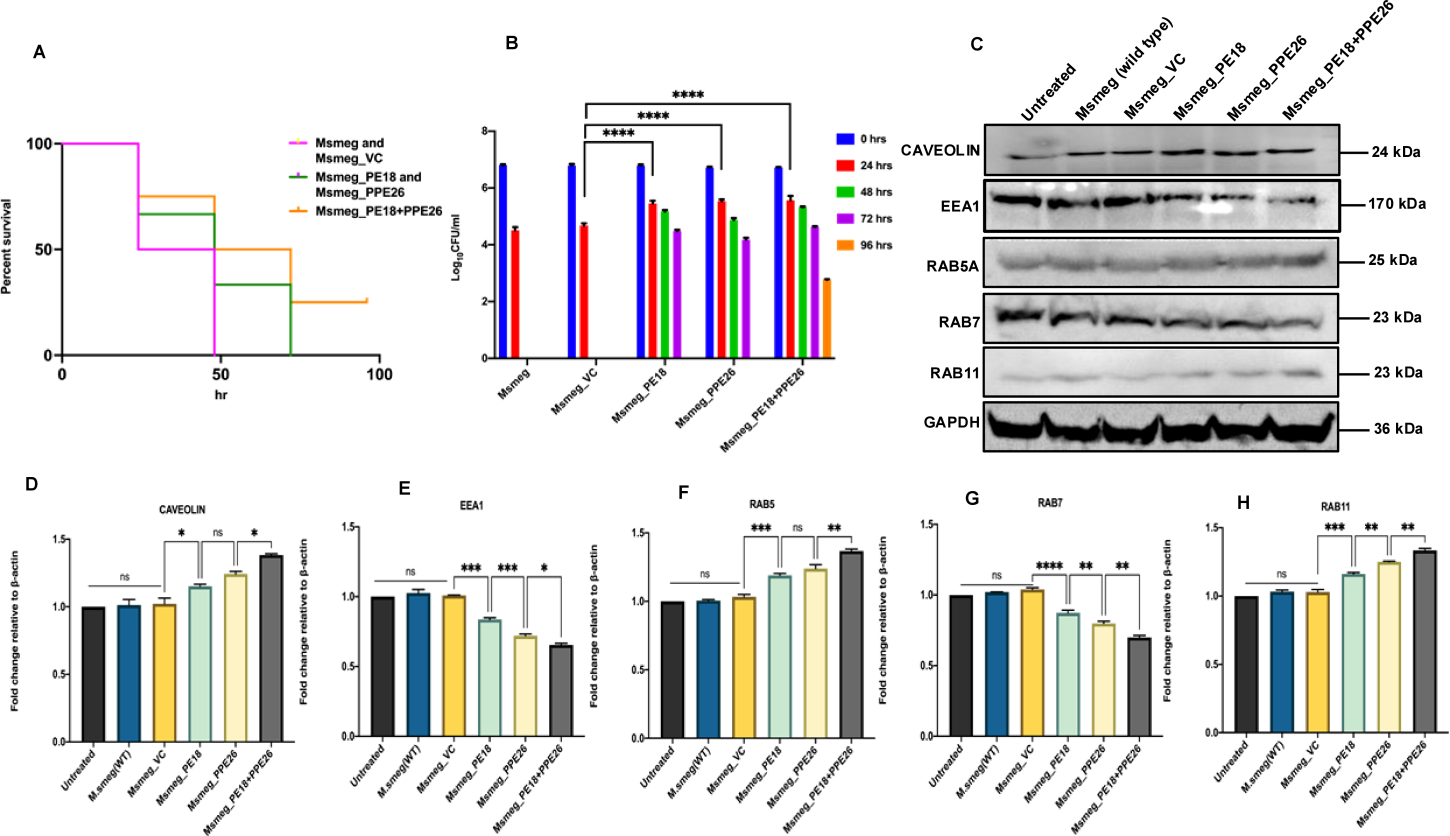
PE18 and PPE26 induce increased survival of recombinant *Msmeg* inside macrophages and likely regulate endosome-phagosome maturation dynamics. **(A, B)** 6-well culture plates were used to seed 2 million RAW264.7 cells/well and left overnight for adherence. *Msmeg*, *Msmeg_VC*, and *Msmeg* expressing PE18, PPE26, and PE18+PPE26 were used to infect macrophages at an MOI of 1:10 for 24, 48, 72, and 96 h. After the completion of infection, 0.01% SDS was used to lyse the cells, followed by plating on 7H11 agar plates. Growth was observed on the fourth day after plating. The CFU assay was used for survival analysis. The survival of *Msmeg* and *Msmeg_VC* inside the macrophages was used as a control. **(C)** Western blots showing the levels of endosomal markers in macrophage cells 24 h after infection with *Msmeg* using αCaveolin, αEEA1, αRab5A, αRab7, and αRab11 antibodies. **(D)** Caveolin protein levels were normalized to GAPDH by densitometric quantification and are shown as a bar graph. **(E)** Densitometric analysis of EEA1 protein levels normalized to GAPDH. **(F)** Densitometric quantification of Rab5 protein levels relative to GAPDH is presented as a bar graph. **(G)** Densitometric quantification of Rab7 protein levels normalized to GAPDH, depicted as a bar graph. **(H)** Densitometric analysis of Rab11 protein levels normalized to GAPDH. Protein levels are shown as [%] relative to GAPDH. Three independent experiments were performed to determine the level of endosome-phagosome maturation markers. Two-way ANOVA was used for data analysis, followed by Tukey’s multiple comparison test. *P values less than0.05, **P values less than0.01, ***P values less than0.001 and, ****P values less than 0.0001 as compared to controls. n. s shows that the data obtained were not significant.

*Msmeg* expressing PE18, PPE26, and PE18+PPE26 showed increased survival inside RAW264.7 cells compared to the wild-type and vector containing *Msmeg* (**Figures 8A, B**). Notably, PE18+PPE26 expressing *Msmeg* exhibited a synergistic effect, as gauged by the comparatively increased survival inside macrophages (up to 96 h) compared to that of PE18 and PPE26 alone (**Figures 8A, B**). These findings show that PE18 and PPE26 function cooperatively to regulate *Mtb* pathogenesis. These observations pointed to manipulation of phagosome maturation, which prompted us to explore the mechanistic details of maturation arrest by PE18 and PPE26 proteins. We infected macrophages with wild-type and recombinant *Msmeg*. Protein samples were prepared 24 h post-infection and western blot analysis was performed using αCaveolin, αEEA1, αRab5A, αRab7, and αRab11 antibodies. We observed an increase in the expression of caveolin upon infection by the recombinants (*Msmeg*_PE18, *Msmeg*_PPE26, and *Msmeg*_PE18+PPE26), suggesting enhancement in caveolin-mediated endocytosis of incoming bacteria by macrophages (**Figures 8C, D**). In contrast, the recombinant *Msmeg* inhibited EEA1 expression in infected macrophages, which is essential for the recruitment of the late endosomal protein Rab7 and hence early to late endosome conversion (**Figures 8C, E**). Further, *Msmeg*_PE18, *Msmeg*_PPE26, and *Msmeg*_PE18+PPE26 recombinants also inhibited the production of the late endosomal maturation marker Rab7 upon infection (**Figures 8C, G**). Moreover, *Msmeg* recombinants induced expression of the early endosomal marker Rab5 in macrophages upon infection (**Figures 8C, F**). The endosome recycling marker Rab11 also increased upon infection of macrophages with recombinant *Msmeg* (**Figures 8C, H**). Collectively, these results strengthen the notion that *Mtb* PE18 and PPE26 likely hamper endosome-phagosome maturation to enhance intracellular survival.

**Figure 9:**
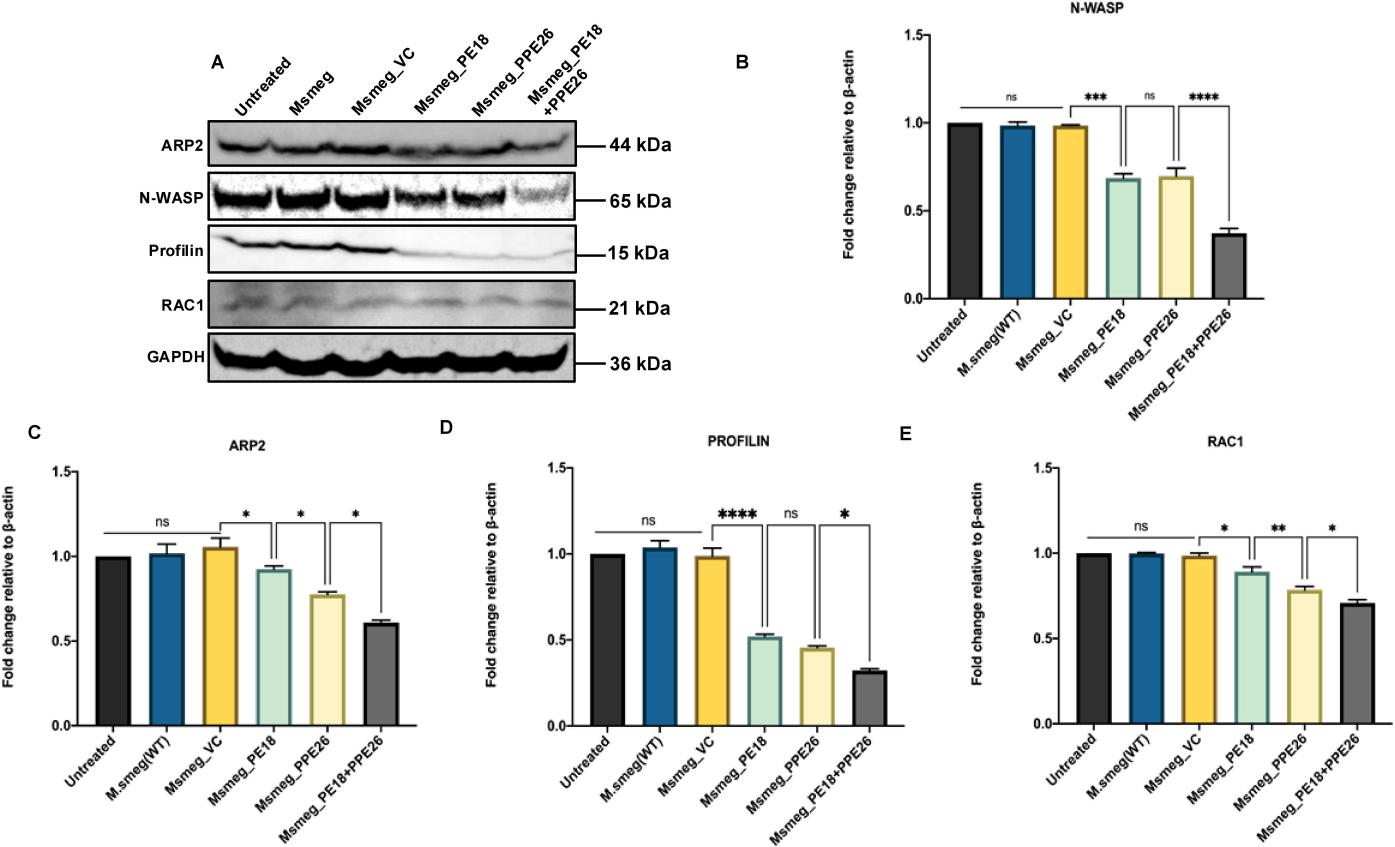
PE18 and PPE26 negatively regulate actin mediated formation of cytoskeletal dynamics. **(A)** Post 24 h after infection of macrophages with wild-type or recombinant *Msmeg*, protein samples were prepared using sample loading buffer. SDS-PAGE was used to run equal amounts of the protein samples. The proteins were then transferred onto PVDF membranes and probed with αArp2, αWasp, αProfilin, and αRac1 antibodies. **(B)** Arp2 protein levels were normalized to GAPDH by densitometric analysis and are shown as a bar graph. **(C)** Wasp protein levels were normalized to GAPDH by densitometric quantification and are shown as a bar graph. **(D)** Profilin protein levels were quantified using densitometry, normalized to GAPDH, and are shown as a bar graph. **(E)** Rac1 protein levels were normalized to GAPDH by densitometric quantification and are shown as a bar graph. UT-, *Msmeg*-, and *Msmeg_ VC-*treated macrophages were used as negative controls. Three independent experiments were performed to determine the level of proteins involved in actin-mediated cytoskeleton dynamics regulation. Two-way ANOVA was used to analyze the data, followed by Tukey’s multiple comparison post-test. *P values less than 0.05, **P values less than 0.01, ***P values less than 0.001 and, ****P values less than 0.0001 compared to controls. n. s shows the results obtained were not significant.

To decipher the possible mechanistic details of endosome-phagosome disruption, we explored the roles of PE18 and PPE26 in regulating actin dynamics. Protein samples were prepared 24 h post-infection and western blot analysis was performed using anti-Arp2, anti-Rac1, anti-Wasp, and anti-Profilin antibodies. *Msmeg*_PE18, *Msmeg*_PPE26, and *Msmeg*_PE18+PPE26 infections strongly suppressed the major regulators of actin polymerization and filamentation, neural Wiskott-Aldrich syndrome protein (N-Wasp), and actin-related protein 2 (ARP2) in macrophages (**Figures 9A–C**). Furthermore, infection with these recombinants also suppressed the expression of Rac1, a Rho-small family GTPase that promotes actin polymerization assembly during filamentation (**Figures 9A, E**). Profilin, an actin-binding protein and recruiter of actin molecules during filament assemblage, was downregulated during infection of macrophages with recombinant *Msmeg* (**Figures 9A, D**). The most pronounced inhibitory effect was observed when macrophages were infected with *Msmeg* expressing PE18 and PPE26 (**Figures 9A–E**). Infection with wild-type *Msmeg* and vector alone served as negative controls. These results imply that PE18 and PPE26 likely hampered the actin dynamics of infected macrophages and showed cooperation in their functions, which might ultimately culminate in the slower kinetics of endosome-phagosome maturation for the efficient survival of *Mtb*.

## 4 Discussion

*Mtb* employs various mechanisms to establish infection and survive within host cells. ESX-5 is a recently evolved genomic locus of *Mtb* that comprises the *pe/ppe* genes *pe18, pe19, ppe25, ppe26*, and *ppe27*, which are involved in virulence. The PE/PPE heterodimeric complex is transported to its effector location in *Mtb* or secreted into the host cell cytosol under infection conditions to potentially modulate host defenses (13). This study showed that PE18 forms a heterodimeric complex with PPE26, and these genes are co-transcribed in *Mtb.* PPE26 also interacts with chaperone EspG5, implying that PE18 and PPE26 form a heterodimeric protein complex recognized by EspG5, which ultimately transports these interacting proteins across the complex cell envelope of *Mtb* to perform a synergistic function. We also unveiled the interactions between PE18 and PPE27, and PE19 and PPE25. The interaction of EspG5 with PPE25 and PPE27 suggests its role in targeting PPE25 and PPE27 to the ESX-5 T7SS transport channel for efficient secretion or cell wall localization. Notably, PE18 forms a heterodimeric complex with both PPE26 and PPE27.

The co-transcription and strong physical association of PE18 and PPE26 suggest functional synergy within *Mtb*. Our findings challenge the previous understanding that each co-operonic PE contains a specific cognate PPE protein. We observed greater flexibility and plasticity in the interactions between PE and PPE proteins, which could potentially be associated with the different phases of the infection process. In addition, we identified interactions between PE18 and PPE27, suggesting the plasticity of these interactions and their potential relevance during different stages of the infection process. The physiological relevance of this switch between partners is gauged to understand its functional importance in virulence and pathogenesis. It has been reported that PE and PPE proteins alone do not exist in soluble form when expressed alone. However, co-expression of these proteins likely stabilizes the heterodimeric complex because of strong physical association (57). Previous studies have revealed that PE and PPE proteins possess intrinsically disordered regions that may contribute to their structural instability (16, 24, 58, 59). However, upon interaction, these regions can transition from a disordered to an ordered state, indicating the importance of protein-protein interactions in stabilizing these complexes. The PE35 and PPE68 proteins mediate the transition from a more disordered to an ordered state upon interaction (24, 58). Notably, we have also shown that PE/PPE proteins PE32 and PPE65, encoded by the ESX-1 locus, are involved in forming heterodimers that exhibit functional synergy in immune modulation (28).

One of the distinctive virulence attributes of *Mtb* is the transport of PE/PPE proteins to cell membrane, cell wall, and cell exterior as secretory proteins (32). Our findings indicate that the PE18 protein is primarily located in the cell wall and secretory fractions of *Mtb*, with only a small amount detected in the cytosolic and cell membrane fractions. In contrast, PPE26 was exclusively localized to the cell wall. The outer cell envelope localization of PE18 and PPE26, along with the secretory nature of PE18, suggests their potential involvement in modulating the host immune response and facilitating interactions between the host and pathogen. Interestingly, we did not detect any ESX-5-associated PPE protein in the secretory fraction. This suggests that these PPE proteins are mainly localized in the outer cell envelope of *Mtb*. This evidence supports the idea that PPE proteins are transported to the outer cell envelope of *Mtb*, where they play crucial roles in immune modulation, host-pathogen interactions, and potentially act as porins involved in importing and exporting small molecules that are essential for the physiological functions of *Mtb* (12, 15, 16, 20, 28, 29). As *Msmeg* was used as a surrogate model to study *Mtb* virulence, the absence of an ESX-5 secretion system in *Msmeg* is likely compensated by utilizing other ESX systems such as ESX-1 and ESX-3. ESX-1, which is involved in the secretion of ESAT-6/CFP-10 and ESX-3 which is primarily involved in iron homeostasis, may facilitate the transport of PE/PPE proteins. Multiple studies have shown that PE/PPE proteins are targeted to the cell envelope when heterologously expressed in *Mmeg*, which supports the notion that alternate secretion mechanisms compensate for the loss of ESX-5 in *Msmeg* (60–68). Multiple other secretion mechanisms are active in *Msmeg*, which can also play roles in the secretion of PE/PPE proteins, such as the SecA2 secretion system and twin arginine translocation (Tat) pathway. Non-canonical mechanisms such as membrane vesicles also enable the release of proteins without traditional signal sequences.

The localization of these proteins at the host-pathogen interface prompted us to examine their role in host immune defense regulation and interaction with the host. We showed that treatment of macrophages with purified PE18, PPE26, and PE18+PPE26 proteins induced the production of pro-inflammatory cytokines and augmented the expression of costimulatory and antigen-presenting molecules in macrophages. Cytokine secretion depends on the innate immune receptor TLR2 and the adapter Myd88. The outcome of innate immune interactions determines the type of adaptive immune response against infection. CD4^+^ T cells are generally believed to provide protective immunity against *Mtb* (69) by recruiting the Th1 cell population at the site of infection (70), although some evidence also suggests a role for CD8+ cells. Infection studies using recombinant *Msmeg* strains expressing *Mtb* PE18 and PPE26 proteins confirmed these findings, demonstrating enhanced production of proinflammatory cytokines such as TNFα, IL6, and IL12. These cytokines play crucial roles in initiating and regulating downstream signalling cascades involved in effector functions of the innate immune system (71). The innate immune receptors TLR2 and TLR4 recognize many of the *Mtb* PE and PPE family proteins - PPE26, PPE57, PPE65, PPE39, PE6, and PE_PGRS5–to initiate downstream signalling cascades for regulating innate and adaptive immunity (26, 28, 72, 73). PE18 and PPE26 are specific TLR2 agonists that regulate host signalling cascades to produce proinflammatory cytokines and the consequent expression of macrophage activation markers to regulate host immunity. Investigating the immunomodulatory effects *in vivo*, we observed that peritoneal macrophages and splenocytes from immunized animals treated with purified proteins exhibited consistent results. Specifically, macrophages activated by PE18 and PPE26 were skewed towards the M1 phenotype, which likely drives the polarization of T-cells towards the Th1 phenotype, thereby amplifying the production of proinflammatory cytokines. The enhanced production of proinflammatory cytokines can induce host tissue damage that *Mtb* utilizes for its successful dissemination (74). Various earlier reports have corroborated the vaccine potential of virulence factors in the PE/PPE family of proteins (34, 75, 76). Immunization with PE18 and PE18+PPE26 proteins resulted in remarkable T-cell activation and an enhanced effector memory phenotype, indicating their potential as vaccine candidates. Though we evaluated the expression of two most widely used and well-established markers for memory cell phenotype, additional markers like CD27, CD127 and CCR7 could have provided a more comprehensive analysis of T cell memory. García-Bengoa et al. (2023) proposed that further investigation is required to characterize mycobacterial PE/PPE proteins, particularly PE18 and PPE26, as immunodominant antigens, which is a highly active research area (77). We also acknowledge the need to explore these proteins further in diverse vaccine formulations. Our findings support their role as immunomodulatory proteins that enhance immune responses by inducing pro-inflammatory cytokines and modulating host actin dynamics, both of which are critical in *Mtb* pathogenicity. Furthermore, previous studies have shown that PE/PPE proteins, especially those associated with the ESX-5 secretion system, play significant roles in *Mtb* virulence and immunogenicity, positioning them as promising vaccine targets (33, 34). These findings suggest that PE18 and PPE26 may be included as part of a multi-antigenic vaccine formulation that could improve their protective potential.

Further, we were intrigued by the observation that recombinant *Msmeg* expressing PE18, PPE26, and PE18+PPE26 exhibited increased survival within macrophages. Notably, the most pronounced intracellular survival was observed when the PE18 and PPE26 proteins were co-expressed. This suggests a synergistic function of these proteins in facilitating intracellular survival.

*Mtb* is resilient and exceptionally efficient in surviving within host macrophages by manipulating its function (9, 27, 78–80). Many PE/PPE proteins have been reported to regulate macrophage function which result in enhanced survival of *Mtb* inside macrophages (11, 63). The survival of *Mtb* within macrophages depends upon the virulence factor-mediated tug-of-war between the pathogen and the host-directed immune defenses (81). *Mtb* infection of macrophages usually manipulates the endosome-phagosome pathway and impairs the maturation of endosomes and phagosomes, consequently preventing phagosome fusion with the lysosome (82, 83). Our results indicate that infection with recombinant *Msmeg* expressing PE18, PPE26, and PE18+PPE26 likely led to inhibition of endosome-phagosome maturation. This inhibition was possibly through the reduced expression of key endosome-phagosome markers such as EEA1 and Rab7. We also observed that PE18, PPE26, and PE18+PPE26 increased caveolin levels used by the host to form cavities during phagocytosis. Rab5A, an early endosome marker that functions together with EEA1, was significantly increased under the influence of these proteins. Conversely, EEA1 protein levels were negatively regulated after infection with recombinant *Msmeg*. EEA1 is required for Rab5 to Rab7 exchange, mediating early endosome conversion into the late endosome (84). The reduced level of Rab7 after infection with recombinant *Msmeg* indicated that PE18 and PPE26 likely arrested endosome-phagosome maturation. Interestingly, we found increased levels of Rab11, a marker of recycling endosomes, regulated by PE18 and PPE26, suggesting that PE18 and PPE26 efficiently recycled the innate immune receptor TLR2 on the macrophage surface for efficient initiation of downstream signaling cascades to hamper host-directed defense strategies. Overall, these findings indicate that the presence of PE18, PPE26, and the combination of PE18 and PPE26 hinders the normal progression of endosome-phagosome maturation processes. Though immunofluorescence staining of these markers could have complemented our results by providing comprehensive insights into the spatial dynamics of endosome and phagosome maturation processes, the expression validation of key markers indicated endosome-phagosome dysregulation. Further validation through immunofluorescence staining for colocalization of these markers could authenticate whether these proteins are found in the same compartments and how recombinant *Msmeg* inhibits the process of endosome-phagosome maturation.

The lag in converting the early endosome to the late endosome is attributed to the downregulation of actin polymerization or F-actin formation (85). Therefore, we examined the actin dynamics of macrophages after infection with *Msmeg* containing PE18, PPE26, and PE18+PPE26. The molecular basis of actin polymerization is tightly regulated by the catalytic subunit, which regulates the rapid conversion of monomeric globular actin (G-actin) to F-actin, forming the cytoskeleton (86). Rac1 is a small GTPase that performs essential roles in actin polymerization and activates nucleation-promoting factors (NPF), including N-Wasp and Wave 2 (87). These nucleation factors ultimately activate the proteins involved in actin branching and formation of long non-branched filaments, including Arp2/3 and Profilin, respectively. Profilin is a canonical regulator of actin polymerization (88). G-actin monomers undergo ADP ribosylation, nucleation, and branched/long actin filament formation (89). Notably, we found decreased levels of Rac1, a GTPase that functions in the activation of nucleation-promoting factors. N-Wasp is recruited as a nucleation-promoting factor that activate Arp2. The complex then binds to G-actin and facilitates the formation of branched F-actin filaments. Intriguingly, we observed decreased levels of N-Wasp and Arp2 mediated by PE18, PPE26, and PE18+PPE26. Moreover, PE18 and PPE26 inhibited the production of profilin, a small protein that interacts with G-actin and nucleates it to form long unbranched cytoskeletal filaments. Taken together, these findings suggest that PE18 and PPE26 of *Mtb* inhibit actin-mediated cytoskeletal dynamics that likely hampers the efficient maturation of endosomes and phagosomes, potentially leading to impaired pathogen clearance. The immunofluorescence staining could have further validated the expression and localization of these markers to offer complementary spatial insights and validation of our results. Nonetheless, western blotting provided precise quantitative data on the expression levels of these crucial actin-regulatory proteins. This approach allowed us to assess their overall abundance and infer potential impacts on actin dynamics and phagosome maturation. These intriguing preliminary findings suggest a previously unknown role for PE18/PPE26 proteins in attenuating host immune defenses, thereby promoting the efficient intracellular survival of *Mtb* and ultimately enhancing its virulence and pathogenesis.

**Figure 10:**
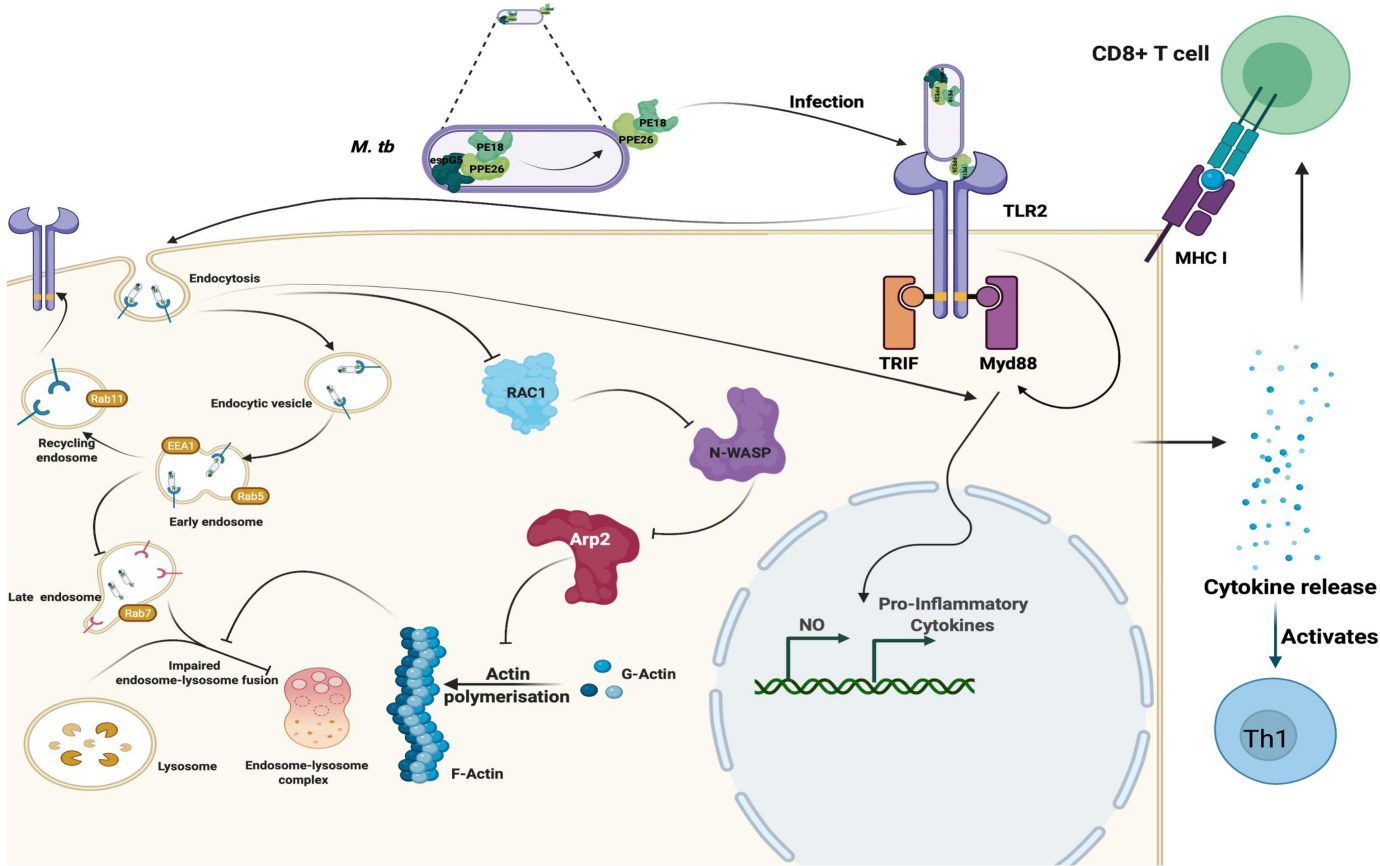
*Mtb* PE18 and PPE26 employ TLR2 and Myd88 for regulating immune response and endosome-phagosome maturation: A Model. *Mtb* PE18 and PPE26 heterodimers interact with the innate immune receptor TLR2, recruiting adapter Myd88 to initiating downstream signaling cascades and the consequent secretion of proinflammatory cytokines and NO. PE18 and PPE26 primed peritoneal macrophages and splenocytes showed increased expression of activation markers, proinflammatory cytokine production, and skewed memory response towards T-cells cell-mediated effector memory. PE18 and PPE26 proteins inhibit actin-mediated cytoskeleton dynamics by regulating the protein levels of Arp2, N-Wasp, Profilin, and Rac1. In addition to the regulation of actin dynamics, PE18 and PPE26 possibly hampers endosome-phagosome maturation by modulating the early, late, and recycling endosome markers EEA1, Rab5, Rab7, and Rab11. These findings highlight that *Mtb* PE18 and PPE26 mediated subversion of innate defenses leads to efficient survival and virulence.

The findings that PE18 and PPE26 induce the production of proinflammatory cytokines and activation of macrophages, along with the activation of CD4+ and CD8+ T cells as well as dysregulation of phagosome-lysosome maturation, suggest that *Mtb* exploits these proteins to hinder phagolysosome maturation as a tactic to survive and replicate inside macrophage cells. Moreover, the enhanced production of proinflammatory cytokines by *Mtb* damages host cells and facilitates dissemination when the bacterial burden is high. Based on these novel observations, we propose a model (**Figure 10**) to depict the mechanistic details by which *Mtb* PE18 and PPE26 perform their functions. The interaction between PE18 and PPE26, forming a heterodimeric complex, plays a key role in the activation of TLR2, an innate receptor present on macrophages. This interaction triggers downstream signaling events that involve the recruitment of MyD88 and the subsequent induction of proinflammatory cytokines. PE18 and PPE26 can activate macrophages, as evidenced by the increased production of activation markers such as CD80, MHC class I, and MHC class II molecules. In addition, these proteins demonstrate immunogenicity *in vivo*, promoting a Th1 immune response and enhancing the generation of effector memory T-cells.

An intriguing observation, though preliminary that remains to be further validated, from our study is that *Mtb* PE18 and PPE26 exert inhibitory effects on actin-mediated cytoskeletal dynamics, which ultimately govern the maturation of endosomes and phagosomes. This inhibition possibly affects the fusion of phagosomes with lysosomes, thereby enabling the successful survival of *Mtb* within macrophages. These findings point towards a novel and crucial virulence strategy employed by *Mtb*, utilizing the PE/PPE family proteins, PE18 and PPE26, to facilitate persistent replication within macrophages and enhance overall virulence and pathogenesis. The multifaceted strategies employed by *Mtb*, involving the enigmatic PE/PPE proteins, underscore the extensive arsenal of utilization by the bacterium.

## Data Availability

The original contributions presented in the study are included in the article/**Supplementary Material**. Further inquiries can be directed to the corresponding authors.

## Supplementary material

Supplementary Figure 1 Purification profile of *Mtb* PE18, PPE25, PPE26, and PPE27. **(A-D)** PE18, PPE25, PPE26, and PPE27 proteins were purified by Ni-NTA affinity chromatography. After purification, the samples were prepared in sodium dodecyl sulfate (SDS) sample loading buffer. Proteins were separated using SDS-PAGE and stained with Coomassie Brilliant Blue. The purity of the purified protein was validated using an anti-His antibody. The sizes of the protein bands are shown in the figure.

Supplementary Figure 2 Validation of recombinant *Msmeg* strains containing PE18, PPE26, and PE18+PPE26 proteins using protein-specific antibodies. **(A)** *Msmeg* competent cells were used for electroporation. One microgram of pST-2K vector, *pe6*, *ppe26*, and *pe18+ppe26* containing plasmid DNA was electroporated. After the revival of electroporated *Msmeg*, the cells were plated on 7H11 agar plates containing antibiotics. Growth was observed on the fourth day of plating. Single colonies were grown in 7H9 liquid media, and after three days of growth, 30 ml of secondary culture was started and grown until OD600nm reached 0.6. Protein lysates were prepared by lysing the cells. Thirty micrograms of total protein were separated by SDS-PAGE, and Western blotting was performed using αPE18 (1:1000) and αPPE26 (1:1000) antibodies. Pre-immune sera were used as negative controls. The sizes of the protein bands and antibodies used in western blotting are shown in the figure. Arrows show PPE26 protein expressed in recombinant *Msmeg*. **(B)** Western blotting showing the specificity of anti-PE18, anti-PPE25, anti-PPE26, and anti-PPE27 antibodies using purified proteins. The sizes of the protein bands and antibodies used are shown in the figure.

Supplementary Figure 3
*In-silico* prediction of protein-protein interactions among ESX-5 encoded PE/PPE and chaperone EspG5. **(A–C)** ESX-5 encoded PE proteins PE18 and PE19; PPE proteins PPE25, PPE26, and PPE27; and chaperone EspG5 were modeled and docked using the ClusPro docking server (https://cluspro.org). The STRING database (http://string-db.org) was used to determine the functional protein association networks. Molecular docking and STRING analysis revealed interactions between PE18-PPE26-EspG5, P18-PPE25-EspG5, and PE19-PPE26-EspG5.

Supplementary Figure 4 PE proteins PE18 and PE19 form homodimer. **(A, B)** *pe18* and *ppe19* were cloned into pET-28a and pGEX-6P2 vectors to express His-tagged and GST-tagged proteins. Proteins were coexpressed in *E. coli* (DE3) cells, cell lysates were prepared, and a His-pull-down assay was performed. After elution of protein complexes, samples were prepared in loading buffer, separated using SDS-PAGE, and subjected to western blotting with αHis and αGST antibodies. The expression of GST-only protein with His-tagged PE18 and PE19 was used as a negative control. **(C–E)** PE18, PPE26, and PE18+PPE26 (1:1 molar ratio) purified proteins were analyzed for their secondary structural properties using Circular Dichroism.

Supplementary Figure 5 PE18 and PPE26 induced the expression of costimulatory molecule CD80 in macrophages. Peritoneal macrophages were isolated from immunized mice treated with the PE18, PPE26, and PE18+PPE26 proteins. The expression of CD80 costimulatory molecules on peritoneal macrophages was determined using FACS analysis. The APC-linked anti-CD80 monoclonal antibody was used to stain the cells and is represented as contour plots. Non-restimulated (89) and LPS stimulated cells were used as controls. Macrophages isolated from the peritoneum of PBS-treated mice were used as controls. PS (protein restimulated).

Supplementary Figure 6 PE/PPE proteins PE18 and PPE26 evoked production of the macrophage activation marker MHCI. Peritoneal macrophages were harvested from immunized mice and stimulated with purified PE18, PPE26, or PE18+PPE26 proteins for 24 h to determine the macrophage activation marker MHC1. FACS was used to determine the expression level of MHCI using an APC-CY7 conjugated anti-MHCI monoclonal antibody and is represented as contour plots. Non-restimulated (89) and LPS stimulated cells were used as controls. Macrophages isolated from the peritoneum of PBS-treated mice were used as controls. PS (protein restimulated).

Supplementary Figure 7 PE18 and PPE26 elicited expression of macrophage activation marker MHCII. Increased expression of the macrophage activation marker MHCI in peritoneal macrophages isolated from immunized mice and stimulated with recombinant purified PE18, PPE26, and PE18+PPE26 at 24 h post-stimulation. The PE-CY7 conjugated anti-MHCII monoclonal antibody was used to analyze the MHCII expression level on the macrophage surface using FACS analysis and is represented as contour plots. Non-restimulated (89) and LPS stimulated cells were used as controls. Macrophages isolated from the peritoneum of PBS-treated mice were used as controls. PS (protein restimulated).

Supplementary Figure 8 PE/PPE proteins PE18 and PPE26 regulate T-cell memory responses. Splenocytes were isolated from protein-immunized and PBS-treated mice and were cultured *in vitro.* Cells were stimulated with PE18, PPE26, and PE18+PPE26 proteins. Non-restimulated splenocytes and splenocytes isolated from PBS-treated mice were used as the controls. The cells were stained with αCD3, αCD4, αCD8, αCD44, and αCD62L antibodies conjugated to a fluorophore. FACS analysis was performed, and data were collected and analyzed using FlowJo™10. **(A)** Expression levels of CD44 and CD62L memory markers in CD4+ T-cells. **(B)** Expression levels of CD44 and CD62L memory markers in CD8+ T cells. The proteins used in the treatment and control groups are shown in figure. NS (non-restimulated), PS (protein restimulated).

Supplementary Figure 9
*Mtb* PE18 and PPE26 proteins preferentially activate CD8+ T-cell expansion. Splenocytes derived from PE18-, PPE26-, and PE18+PPE26 immunized mice-derived splenocytes were isolated. The cells were cultured *in vitro*, and the numbers of CD4+ and CD8+ T cells were determined under protein non-stimulating and stimulating conditions. Cells were analyzed by FACS using fluorophore-tagged αCD3, αCD4, and αCD8 antibodies and are represented as dot plots. NS (non-restimulated), PS (protein restimulated).
