# Supplementary material for: Deciphering the functional roles of PE18 and PPE26 proteins in modulating Mycobacterium tuberculosis pathogenesis and immune response
Source: Front Immunol. 2025 Jan 30;16:1517822. doi: 10.3389/fimmu.2025.1517822 (PMC11821933; doi:10.3389/fimmu.2025.1517822)
Supplement: Supplementary Figure 1 — Purification profile of Mtb PE18, PPE25, PPE26, and PPE27. (A-D) PE18, PPE25, PPE26, and PPE27 proteins were purified by Ni-NTA affinity chromatography. After purification, the samples were prepared in sodium dodecyl sulfate (SDS) sample loading buffer. Proteins were separated using SDS-PAGE and stained with Coomassie Brilliant Blue. The purity of the purified protein was validated using an anti-His antibody. The sizes of the protein bands are shown in the figure. [file DataSheet1.pdf]

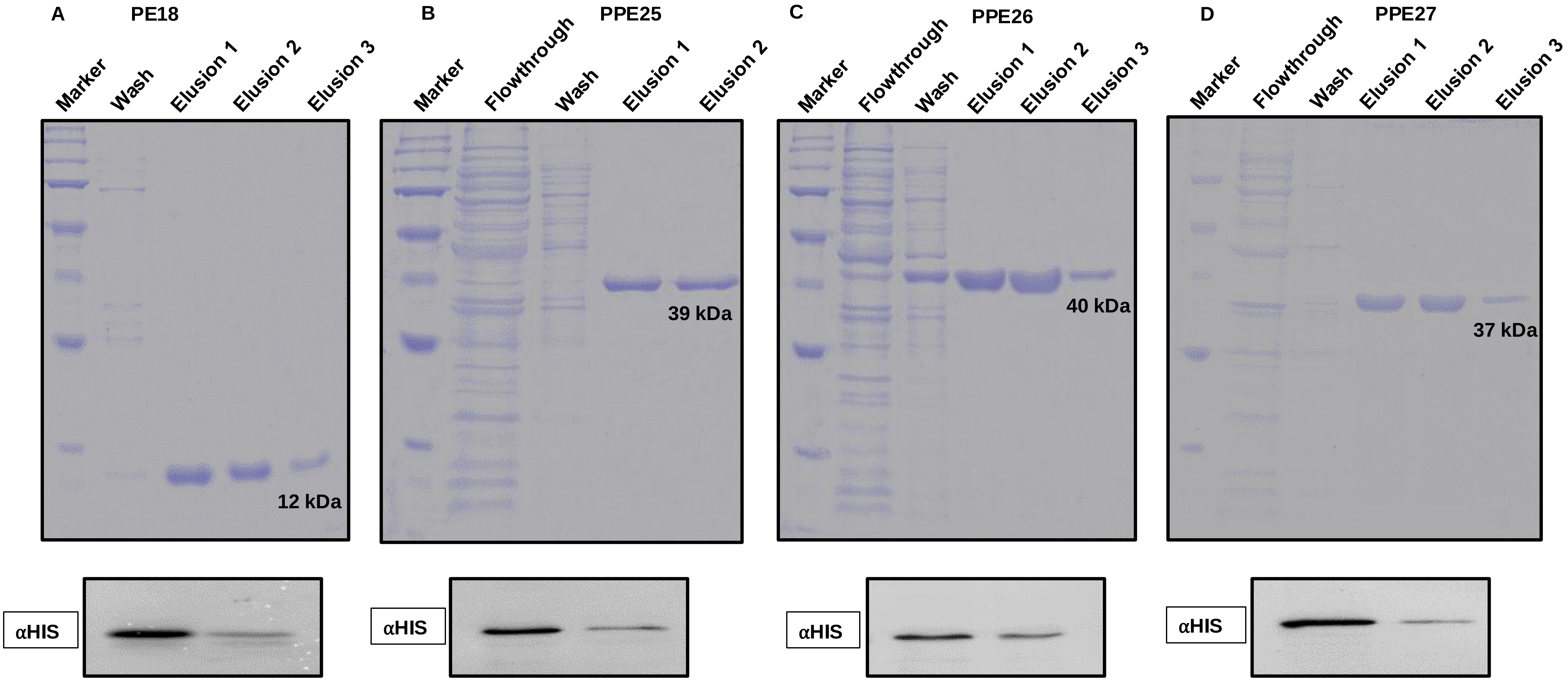
A
PE18
Marker
Wash
Elusion 1
Elusion 2
Elusion 3
12 kDa
αHIS
B
PPE25
Marker
Flowthrough
Wash
Elusion 1
Elusion 2
39 kDa
αHIS
C
PPE26
Marker
Flowthrough
Wash
Elusion 1
Elusion 2
Elusion 3
40 kDa
αHIS
D
PPE27
Marker
Flowthrough
Wash
Elusion 1
Elusion 2
Elusion 3
37 kDa
αHIS

Figure S1

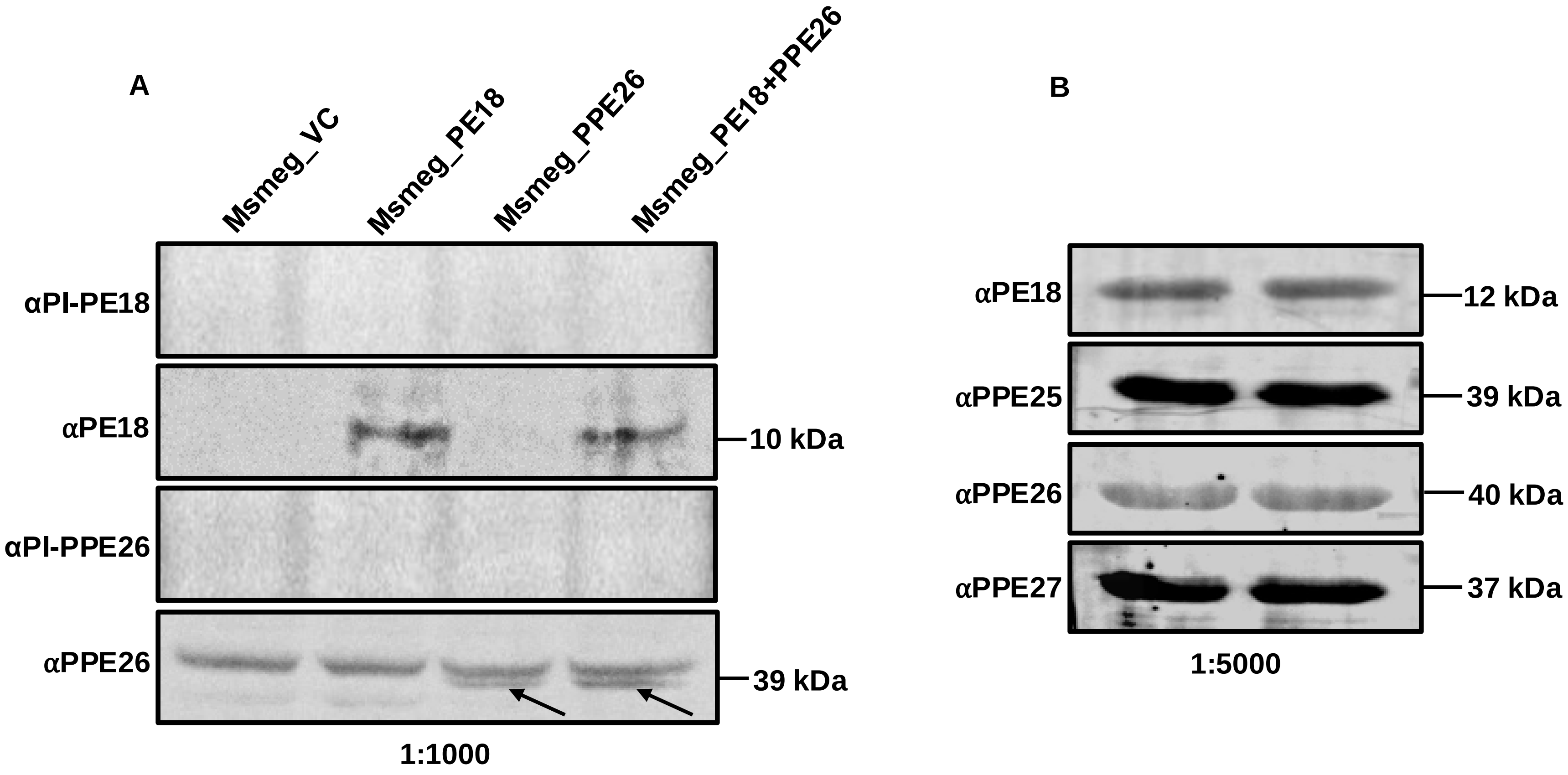
A
Msmeg_VC
Msmeg_PE18
Msmeg_PPE26
Msmeg_PE18+PPE26
αPI-PE18
αPE18
10 kDa
αPI-PPE26
αPPE26
39 kDa
1:1000
B
αPE18
12 kDa
αPPE25
39 kDa
αPPE26
40 kDa
αPPE27
37 kDa
1:5000

Figure S2

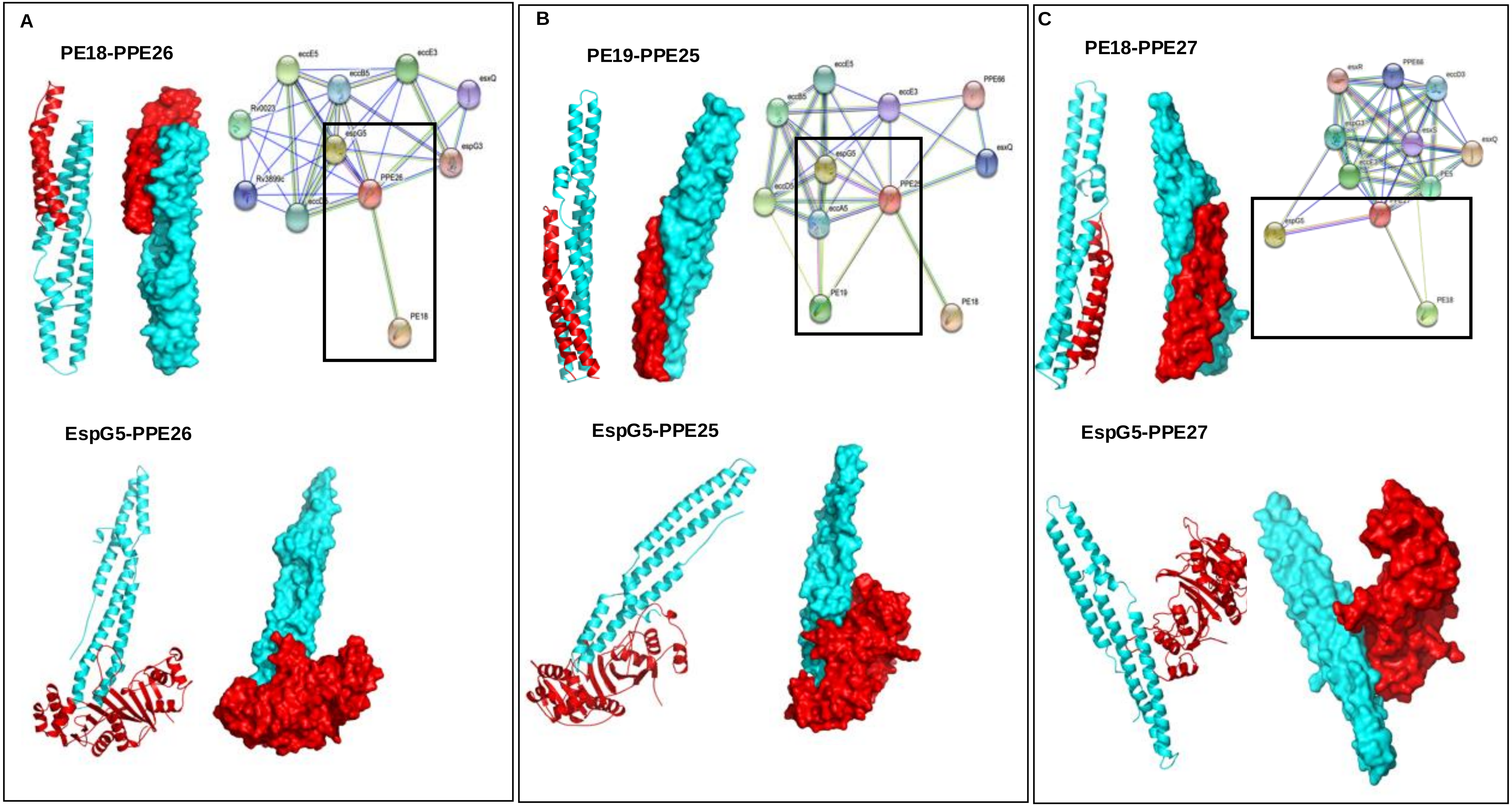
A
PE18-PPE26
EspG5-PPE26
B
PE19-PPE25
EspG5-PPE25
C
PE18-PPE27
EspG5-PPE27

Figure S3

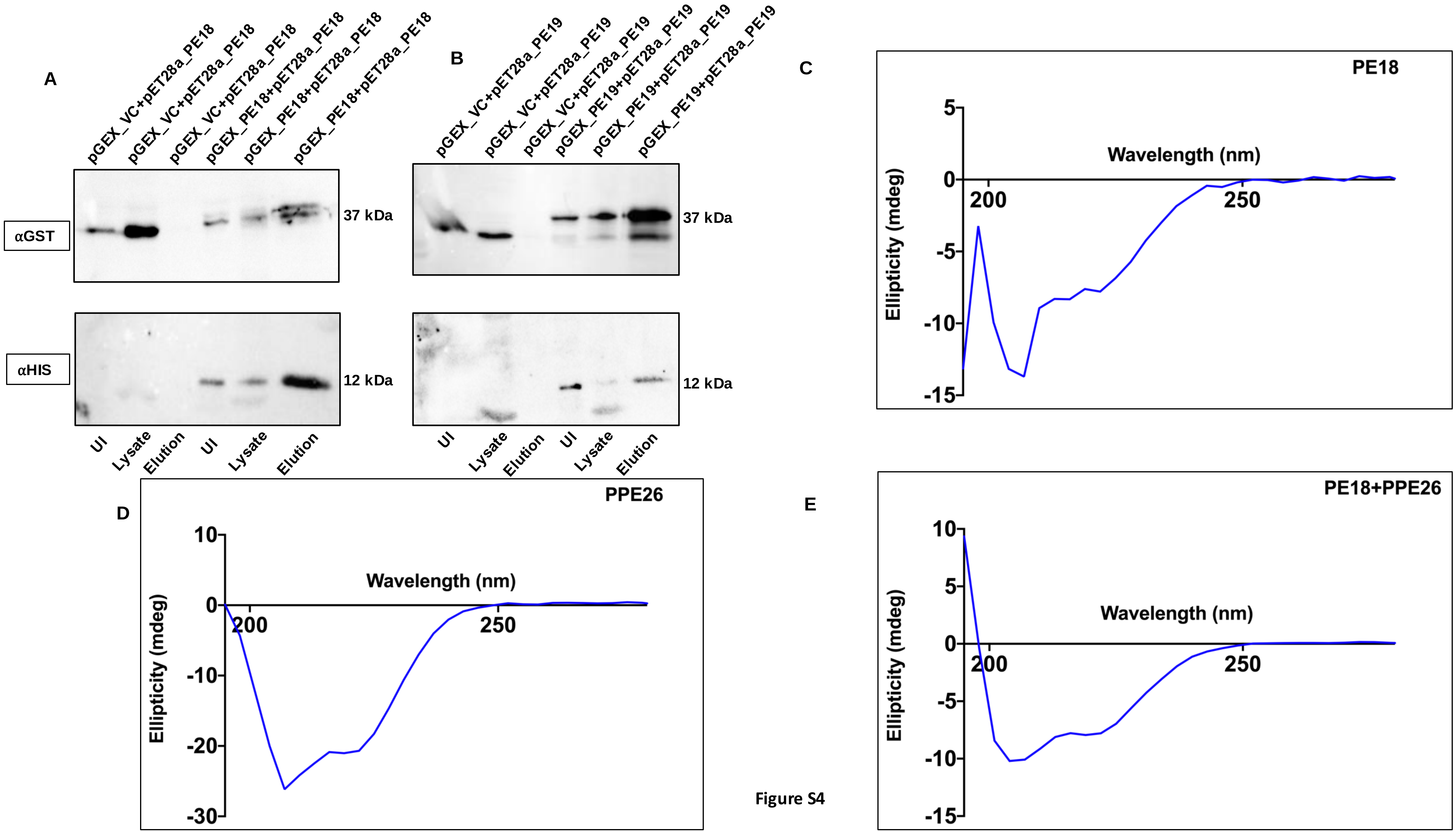
A
pGEX_VC+pET28a_PE18
pGEX_VC+pET28a_PE18
pGEX_VC+pET28a_PE18
pGEX_PE18+pET28a_PE18
pGEX_PE18+pET28a_PE18
pGEX_PE18+pET28a_PE18
αGST
37 kDa
αHIS
12 kDa
UI
Lysate
Elution
UI
Lysate
Elution
B
pGEX_VC+pET28a_PE19
pGEX_VC+pET28a_PE19
pGEX_VC+pET28a_PE19
pGEX_PE19+pET28a_PE19
pGEX_PE19+pET28a_PE19
pGEX_PE19+pET28a_PE19
37 kDa
12 kDa
UI
Lysate
Elution
UI
Lysate
Elution
C
PE18
Wavelength (nm)
Ellipticity (mdeg)
5
0
-5
-10
-15
200
250
D
PPE26
Wavelength (nm)
Ellipticity (mdeg)
10
0
-10
-20
-30
200
250
E
PE18+PPE26
Wavelength (nm)
Ellipticity (mdeg)
10
5
0
-5
-10
-15
200
250

Figure S4

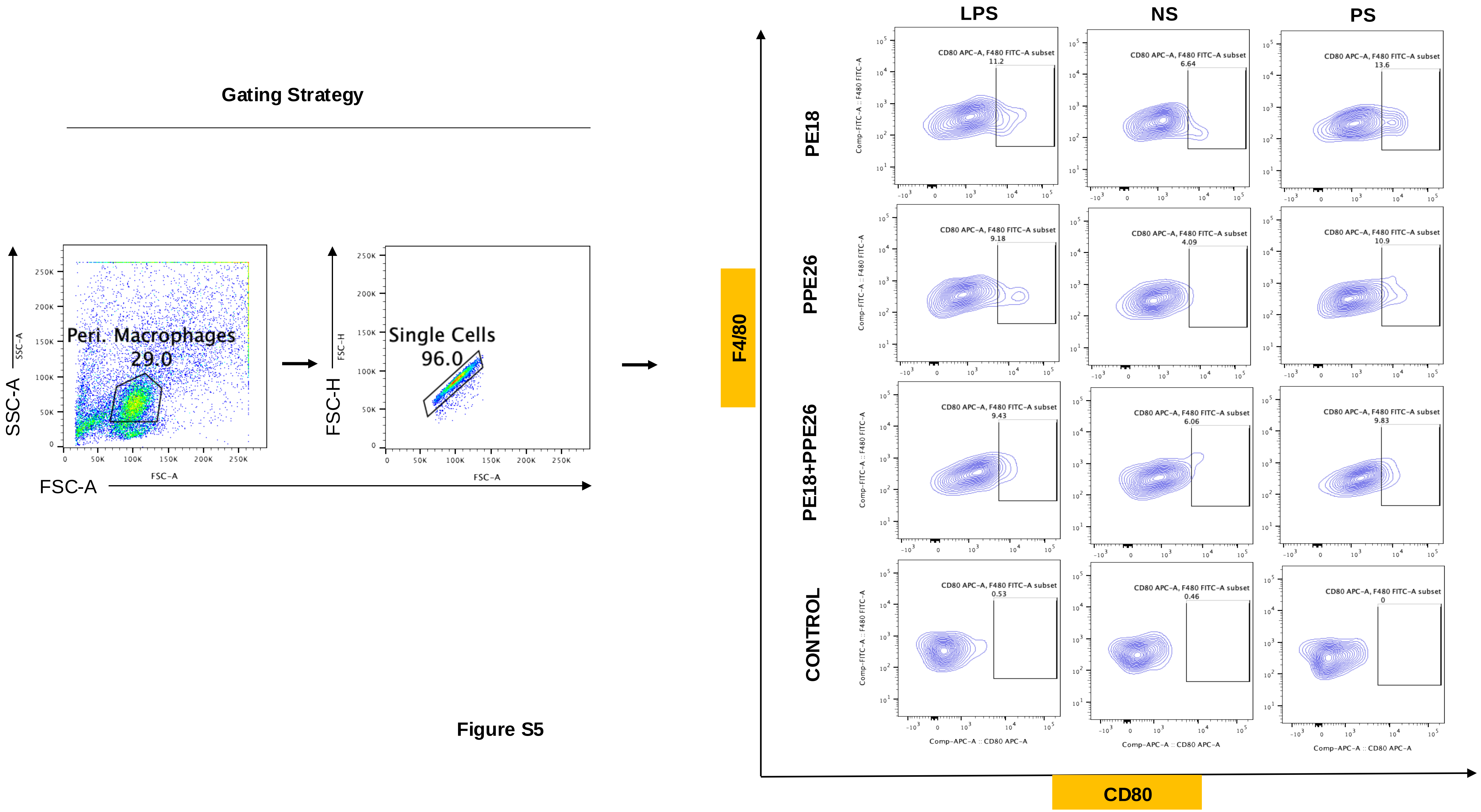
Gating Strategy
Peri. Macrophages
29.0
Single Cells
96.0
SSC-A
FSC-H
FSC-A
LPS
NS
PS
PE18
PPE26
PE18+PPE26
CONTROL
F4/80
CD80
CD80 APC-A, F480 FITC-A subset
11.2
6.64
13.6
9.18
4.09
10.9
9.43
6.06
9.83
0.53
0.46
0
Comp-FITC-A :: F480 FITC-A
Comp-APC-A :: CD80 APC-A

Figure S5

Figure S6

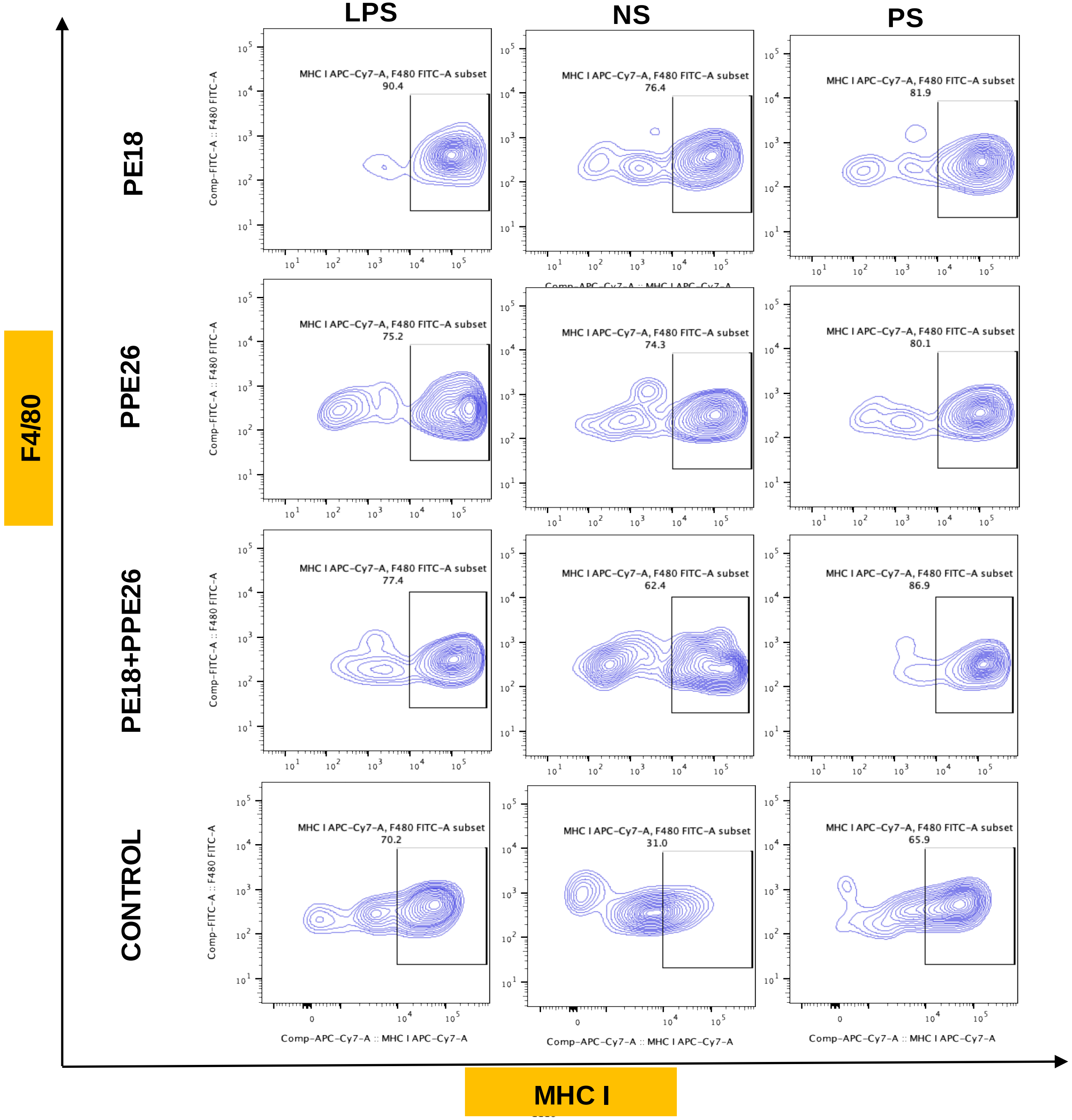
LPS
NS
PS
PE18
PPE26
PE18+PPE26
CONTROL
F4/80
MHC I
MHC I APC-Cy7-A, F480 FITC-A subset
90.4
76.4
81.9
75.2
74.3
80.1
77.4
62.4
86.9
70.2
31.0
65.9
Comp-FITC-A :: F480 FITC-A
Comp-APC-Cy7-A :: MHC I APC-Cy7-A

Figure S7

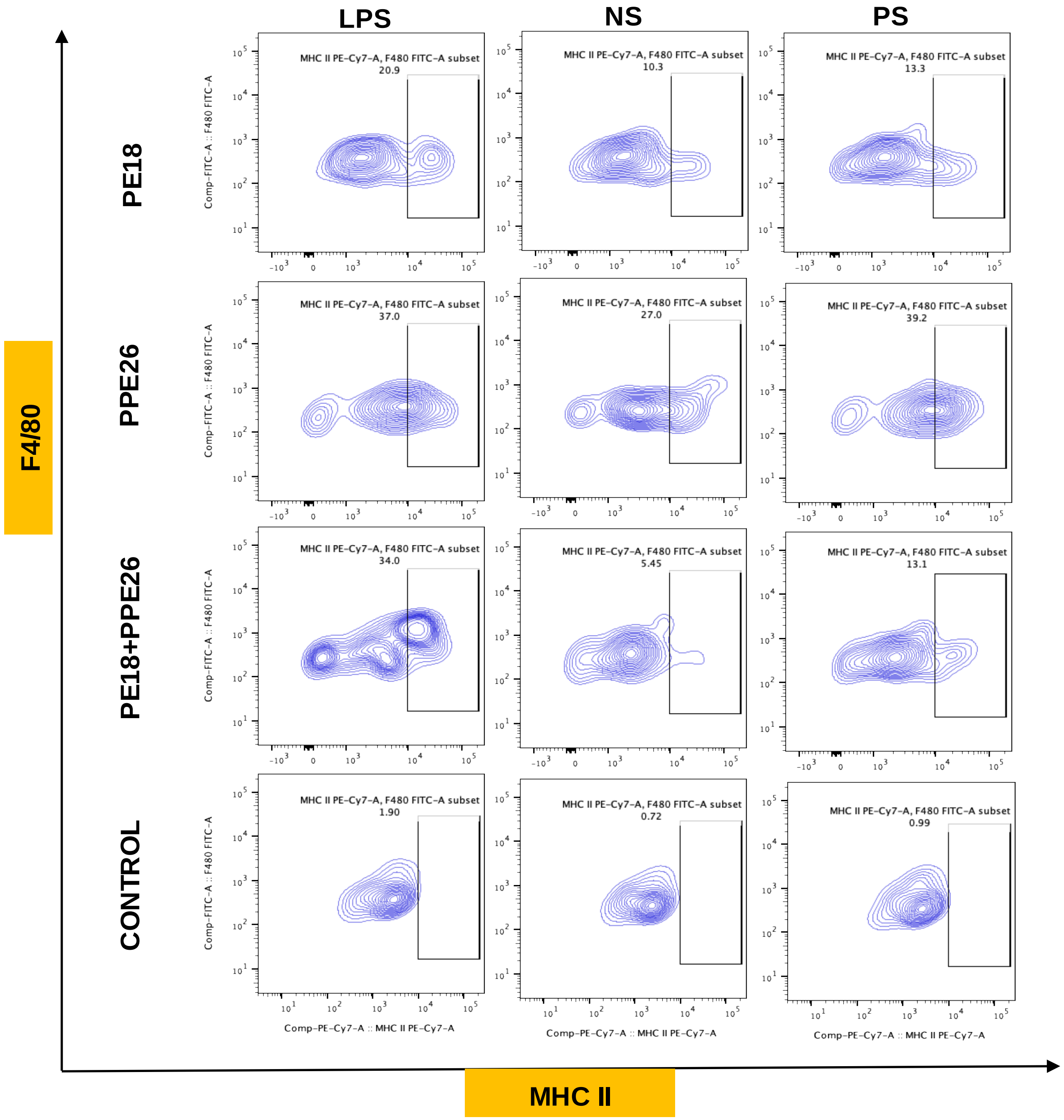

LPS
NS
PS
PE18
PPE26
PE18+PPE26
CONTROL
F4/80
MHC II
MHC II PE-Cy7-A, F480 FITC-A subset
20.9
10.3
13.3
37.0
27.0
39.2
34.0
5.45
13.1
1.90
0.72
0.99
Comp-FITC-A :: F480 FITC-A
Comp-PE-Cy7-A :: MHC II PE-Cy7-A

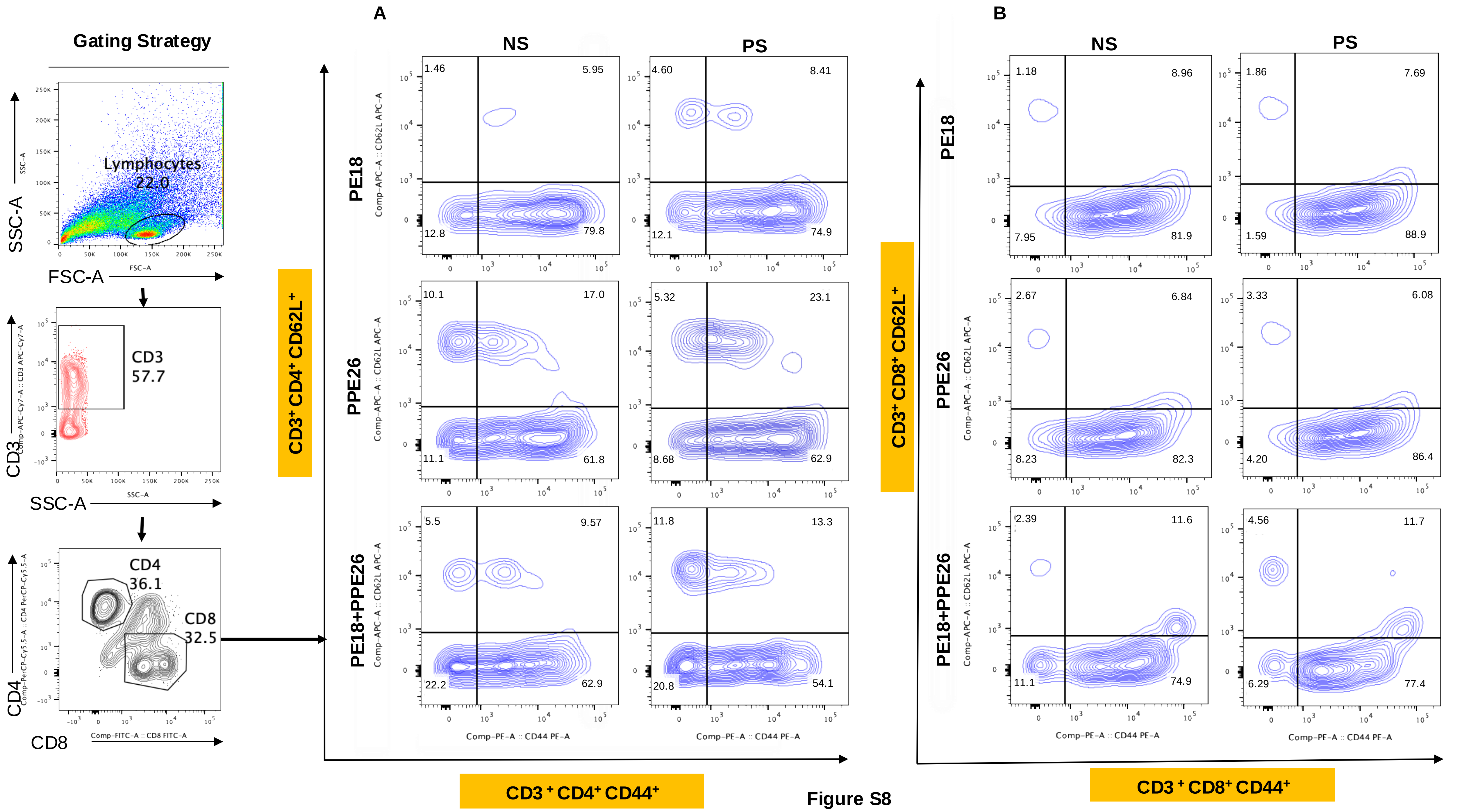

Gating Strategy
Lymphocytes 22.0
SSC-A
FSC-A
CD3 57.7
CD3
SSC-A
CD4 36.1
CD8 32.5
CD4
CD8
A
NS
PS
PE18
PPE26
PE18+PPE26
CD3+ CD4+ CD62L+
CD3+ CD4+ CD44+
1.46
5.95
12.8
79.8
4.60
8.41
12.1
74.9
10.1
17.0
11.1
61.8
5.32
23.1
8.68
62.9
5.5
9.57
22.2
62.9
11.8
13.3
20.8
54.1
Comp-APC-A :: CD62L APC-A
Comp-PE-A :: CD44 PE-A
B
CD3+ CD8+ CD62L+
CD3+ CD8+ CD44+
1.18
8.96
7.95
81.9
1.86
7.69
1.59
88.9
2.67
6.84
8.23
82.3
3.33
6.08
4.20
86.4
2.39
11.6
11.1
74.9
4.56
11.7
6.29
77.4


Figure S8

Figure S9

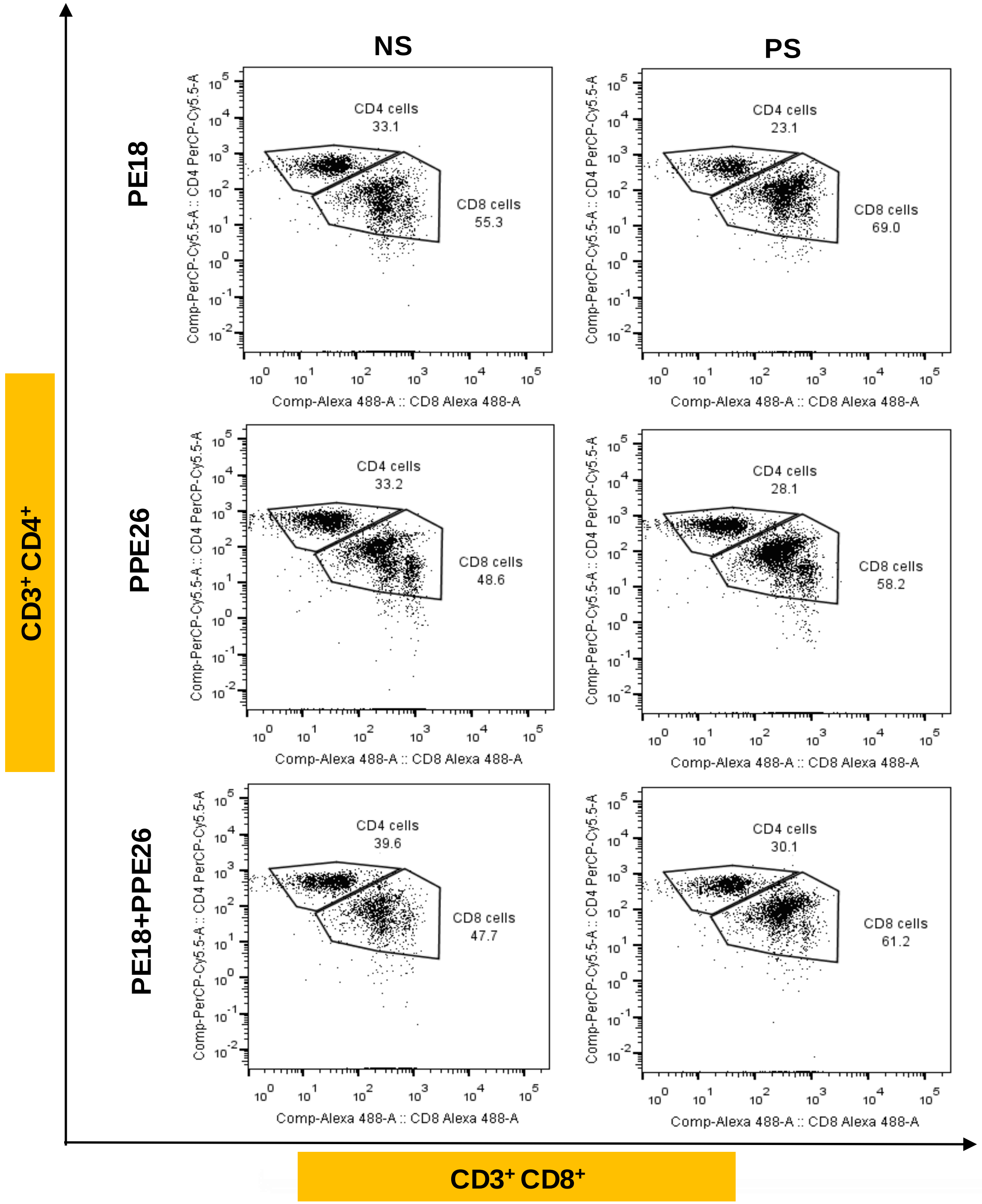
NS
PS
PE18
PPE26
PE18+PPE26
CD4 cells
33.1
CD8 cells
55.3
CD4 cells
23.1
CD8 cells
69.0
CD4 cells
33.2
CD8 cells
48.6
CD4 cells
28.1
CD8 cells
58.2
CD4 cells
39.6
CD8 cells
47.7
CD4 cells
30.1
CD8 cells
61.2
Comp-PerCP-Cy5.5-A :: CD4 PerCP-Cy5.5-A
Comp-Alexa 488-A :: CD8 Alexa 488-A
CD3+ CD4+
CD3+ CD8+
