# Supplementary material for: Deciphering the functional roles of PE18 and PPE26 proteins in modulating Mycobacterium tuberculosis pathogenesis and immune response
Source: Front Immunol. 2025 Jan 30;16:1517822. doi: 10.3389/fimmu.2025.1517822 (PMC11821933; doi:10.3389/fimmu.2025.1517822)
Supplement: Supplementary file 2 [file Table1.docx]

| **Gene_Vector** | **Primer** | **Primer sequence (5’-3’)** |
| --- | --- | --- |
| PE18_pGEX6P2/ PE18_pET28a | Forward | AAAAGGATCCATGTCGTTTGTGACTACCCAACC |
|  | Reverse | AAAAACTCGAGCTAGCCGGCCGCGCC |
| PE19_pGEX6P2/ PE19_pET28a | Forward | AAAAAGGATCCATGTCGTTCGTGACCACACAGC |
|  | Reverse | ATATCTCGAGTCAGCCGGCAGCGGCTGC |
| PPE25_pGEX6P2/ PPE25_pET28a | Forward | AAAAGGATCCTTGGACTTCGGGGCGTTACC |
|  | Reverse | AAAACTCGAGTTATCCGGCCGATGGCGG |
| PPE26_pGEX6P2/ PPE26_pET28a | Forward | AAAAGGATCCATGGATTTTGGGGCGTTGC |
|  | Reverse | AAAACTCGAGCTATCCGGCGAAGGGTGGG |
| PPE27_pGEX6P2/ PPE27_pET28a | Forward | AAAAGGATCCATGGACTTCGGGGCGTTACCGC |
|  | Reverse | AAAACTCGAGCTATCCCGCCGACGGAGACCGG |
| EspG5_pGEX6P2/ EspG5_pET28a | Forward | AAAAGAATTCATGGATCAACAGAGTACCCGCACCGA |
|  | Reverse | AAAACTCGAGTCATACTCTGCTGTGTGTTTTC |
| PE18_pST2K | Forward | AAAAAGAATTCATGTCGTTTGTGACTACCCAAC |
|  | Reverse | ATATAAGCTTCTAGCCGGCCGCGGCCGCGT |
| PPE26_pST2K | Forward | AAAAGGATCCATGGATTTTGGGGCGTTGC |
|  | Reverse | AAAAAAGCTTCTATCCGGCGAAGGGTGGG |
| Intergenic region | Forward | CCGGCGGCCGCCGATGAAGTGT |
|  | Reverse | CGGCAAACGCCGCCTCAAAAGCG |

**Table S1: Vectors and primers used in this study.**
