# Supplementary material for: Deciphering the functional roles of PE18 and PPE26 proteins in modulating Mycobacterium tuberculosis pathogenesis and immune response
Source: Front Immunol. 2025 Jan 30;16:1517822. doi: 10.3389/fimmu.2025.1517822 (PMC11821933; doi:10.3389/fimmu.2025.1517822)
Supplement: Supplementary file 3 [file Table2.docx]

**Table S2:**

1. **Protein-protein docking results and hydrogen bond networks.**

| **Proteins** | **Cluster members** | **Score** |
| --- | --- | --- |
| **PE18-PPE26** | **89** | **-1120.4** |
| **PE18-PPE27** | **94** | **-1387.1** |
| **PE19-PPE25** | **99** | **-1222.6** |
| **EspG5-PPE25** | **118** | **-956.8** |
| **EspG5-PPE26** | **87** | **-806.2** |
| **EspG5-PPE27** | **100** | **-889.0** |

**B. Hydrogen bonding interactions between PE18-PPE26**

| **S. no.** | **PE18** | **Dist. [Å]** | **PPE26** |
| --- | --- | --- | --- |
| 1 | GLN 54 [HE21] | 1.97 | GLU 55 [OE1] |
| 2 | TYR 62 [HH] | 1.80 | TYR 45 [OH] |

**C. Hydrogen bonding interactions between EspG5-PPE26**

| **S. no.** | **EspG5** | **Dist. [Å]** | **PPE26** |
| --- | --- | --- | --- |
| 1 | ARG 109 [HH21] | 1.74 | GLY 44 [O] |
| 2 | ARG 34 [HH21] | 2.25 | TYR 45 [OH] |
| 3 | LYS 235 [HZ1] | 1.92 | GLN 51 [OE1] |
| 4 | ARG 228 [HH12] | 1.86 | GLU 55 [OE2] |
| 5 | GLN 187 [HE22] | 2.38 | GLU 67 [OE2] |
| 6 | GLU 32 [OE2] | 2.45 | GLN 51 [HE22] |
| 7 | GLN 104 [O] | 1.91 | TYR 72 [HH] |

**D. Hydrogen bonding interactions between PE19-PPE25**

| **S. no.** | **PE19** | **Dist. [Å]** | **PPE25** |
| --- | --- | --- | --- |
| 1 | GLN 54 [HE22] | 1.93 | SER 162 [OG] |
| 2 | GLN 68 [HE21] | 1.94 | TYR 72 [OH] |
| 3 | GLU 46 [OE1] | 2.47 | ARG 14 [HH11] |
| 4 | SER 48 [OG ] | 2.20 | TYR 154 [HH] |

**E. Hydrogen bonding interactions between EspG5-PPE25**

| **S. no.** | **EspG5** | **Dist. [Å]** | **PPE25** |
| --- | --- | --- | --- |
| 1 | ARG 36 [HH11] | 1.78 | ASP 2 [OD2] |
| 2 | LYS 184 [HZ2] | 1.76 | ALA 122 [O] |
| 3 | GLN 256 [HE21] | 1.95 | GLN 128 [OE1] |
| 4 | ARG 34 [HH22] | 1.78 | GLU 137 [OE1] |
| 5 | ARG 109 [HH21] | 2.14 | GLU 137 [O] |
| 6 | ARG 109 [HH11] | 1.99 | GLU 137 [O] |
| 7 | ARG 27 [HH12] | 1.79 | GLU 142 [OE1] |
| 8 | ARG 27 [HH11] | 2.03 | GLN 146 [OE1] |
| 9 | ARG 27 [HH21] | 1.78 | GLN 146 [OE1] |
| 10 | VAL 241 [O] | 1.90 | GLN 128 [HE22] |

**F. Hydrogen bonding interactions between PE18-PPE27**

| **S. no.** | **PE18** | **Dist. [Å]** | **PPE27** |
| --- | --- | --- | --- |
| 1 | GLN 28 [HE21] | 2.47 | ALA 30 [O] |
| 2 | GLN 54 [HE22] | 2.31 | SER 162 [O] |
| 3 | ASP 45 [OD1] | 1.79 | ARG 14 [HH11] |
| 4 | ALA 69 [O] | 1.88 | TYR 72 [HH] |

**G. Hydrogen bonding interactions between EspG5-PPE27**

| **S. no.** | **EspG5** | **Dist. [Å]** | **PPE27** |
| --- | --- | --- | --- |
| 1 | GLU 37 [OE1] | 2.23 | GLY 52 [H] |
